# Bridging the gap between non-targeted stable isotope labeling and metabolic flux analysis

**DOI:** 10.1186/s40170-016-0150-z

**Published:** 2016-04-23

**Authors:** Daniel Weindl, Thekla Cordes, Nadia Battello, Sean C. Sapcariu, Xiangyi Dong, Andre Wegner, Karsten Hiller

**Affiliations:** Luxembourg Centre for Systems Biomedicine, University of Luxembourg, 7, Avenue des Hauts Fourneaux, Esch-sur-Alzette, 4362 Luxembourg; Department of Bioengineering, University of California, Gilman Drive, San Diego, La Jolla, 92037 USA

**Keywords:** Stable isotope labeling, Metabolomics, Non-targeted, Flux analysis, Mass isotopomer distribution analysis, NTFD, Cancer, NAT8L, *N*-acetylaspartate

## Abstract

**Background:**

Metabolism gained increasing interest for the understanding of diseases and to pinpoint therapeutic intervention points. However, classical metabolomics techniques only provide a very static view on metabolism. Metabolic flux analysis methods, on the other hand, are highly targeted and require detailed knowledge on metabolism beforehand.

**Results:**

We present a novel workflow to analyze non-targeted metabolome-wide stable isotope labeling data to detect metabolic flux changes in a non-targeted manner. Furthermore, we show how similarity-analysis of isotopic enrichment patterns can be used for pathway contextualization of unidentified compounds. We illustrate our approach with the analysis of changes in cellular metabolism of human adenocarcinoma cells in response to decreased oxygen availability. Starting without a priori knowledge, we detect metabolic flux changes, leading to an increased glutamine contribution to acetyl-CoA production, reveal biosynthesis of *N*-acetylaspartate by *N*-acetyltransferase 8-like (NAT8L) in lung cancer cells and show that *NAT8L* silencing inhibits proliferation of A549, JHH-4, PH5CH8, and BEAS-2B cells.

**Conclusions:**

Differential stable isotope labeling analysis provides qualitative metabolic flux information in a non-targeted manner. Furthermore, similarity analysis of enrichment patterns provides information on metabolically closely related compounds. *N*-acetylaspartate and NAT8L are important players in cancer cell metabolism, a context in which they have not received much attention yet.

**Electronic supplementary material:**

The online version of this article (doi:10.1186/s40170-016-0150-z) contains supplementary material, which is available to authorized users.

## Background

Over the last decades, cellular metabolism gained increasing interest to pinpoint potential therapeutic intervention points to treat complex diseases. Metabolomics research, analyzing changes in metabolite levels, deepened our understanding of cellular metabolism, which led to the discovery of unanticipated metabolites [1] and disease biomarkers [2, 3]. However, metabolite levels alone provide only a very static view on metabolism. For a system understanding of metabolism, the underlying metabolic fluxes are much more important and informative because they provide a much closer functional link to an observed phenotype [4]. Metabolic fluxes through these pathways do not only depend on metabolite concentrations, but are modulated by intricate regulatory mechanisms [5]. For that reason, they cannot be deduced from metabolite levels alone.

To that end metabolic flux analysis techniques such as flux balance analysis (FBA) and ^13^C metabolic flux analysis (^13^C-MFA) have been developed. FBA employs genome-scale metabolic networks [6] and aims to balance cellular influxes and effluxes with an optimal set of intracellular fluxes [7, 8]. On the other hand, ^13^C-MFA uses much smaller metabolic networks, but combines cellular influxes and effluxes with experimental data obtained from stable isotope labeling experiments [9, 10]. Isotopic labeling patterns are usually analyzed by mass spectrometry (MS) in the form of mass isotopomer distributions (MIDs), which are the mass-aggregated relative isotopologue abundances. Since MIDs of metabolites from a given tracer within a given metabolic network are solely a function of the metabolic fluxes, they can be used to estimate these underlying fluxes [11, 12]. Using mathematical optimization techniques, a set of fluxes is determined which can explain the experimentally observed MIDs for a defined metabolic network model [9, 10].

A drawback, that all current metabolic flux analysis techniques have in common, is that they rely on the exact topological knowledge of the metabolic network of interest. However, knowledge of metabolic networks of most organisms is still not comprehensive, as for example recently shown for mammalian macrophages which were found to produce an unanticipated antimicrobial compound [1].

As stated above, MIDs from stable isotope labeling experiments hold metabolic flux information. This is exploited in ^13^C-MFA, but only in a highly targeted manner. However, there are methods available for the non-targeted MID determination of compounds in complex mixtures after either gas chromatography MS (GC-MS) [13, 14] or liquid chromatography MS (LC-MS) analysis [15–17]. These methods do not rely on any biological knowledge, and thus, are able to account for any unanticipated metabolite. To date, only very few studies performed non-targeted MID analyses and obtained novel biological insights [15, 18]. Mostly, non-targeted detection of stable isotope labeling has been applied in a qualitative manner to separate metabolites produced by the cell from analytical background [19–21]. One major problem is still the lack of appropriate tools to extract biological information out of the mass spectrometric data [22]. In particular, studies covering multiple experimental conditions or time-points generate complex data and require proper tools for efficient analysis and visualization of isotope labeling data.

In this article, we present a novel workflow for stable isotope labeling analysis that allows for the non-targeted detection of (1) pathway activity, highlighting unexpected parts of metabolism; (2) relative flux changes or differential pathway activity between conditions; and (3) the pathway contextualization of unidentified compounds and their vicinity to other metabolites. This workflow can be used for data-driven analyses and hypothesis generation which can be tested in subsequent targeted approaches. We illustrate our workflow by analyzing changes in cellular metabolism of human lung cancer cells in response to varying oxygen availability ranging from atmospheric 21 % O_2_ down to 1 % O_2_. Starting without a priori knowledge, we detected metabolic flux changes, which led to an increased glutamine contribution to acetyl-CoA, show that A549 lung cancer cells produce *N*-acetylaspartate, a compound which is well known to have an important function in neuronal tissue, but was until very recently [23] not known to be produced in other tissues, and show that silencing its biosynthetic enzyme NAT8L exerts a negative growth effect.

## Methods

### Chemicals

Stable isotope labeled tracers were bought from Cambridge Isotope Laboratories, all other chemicals were bought from Sigma-Aldrich. All solvents were of grade *Chromasolv* or higher.

### Cell culture & stable isotope labeling

Human lung adenocarcinoma A549 cells (ATCC CCL-185, [24]) were cultivated in D5030 medium (Dulbecco’s modified Eagle’s medium without glucose, glutamine, phenol red, sodium pyruvate and sodium bicarbonate), supplemented with 10 % dialyzed fetal bovine serum and either 12.5 mM glucose, 12.5 mM [1,2-^13^C_2_]-D-glucose and 4 mM glutamine, or with 25 mM glucose, 2 mM glutamine and 2 mM [U-^13^C]-L-glutamine. Cells were seeded into 12-well multi-well plates at a density of 3×10^5^ cells in 0.75 ml growth medium and cultivated at 37°, in an atmosphere with 5 % CO_2_ and 95 % air with oxygen levels of 1, 5, 10, 15, and 21 %. Before applying the tracer-containing medium for 24 h, cells and media were equilibrated to the respective oxygen levels for 24 h. Under each condition, 3–4 wells were used for metabolite extraction.

### Metabolite extraction and GC-MS analysis

Intracellular metabolites were extracted and polar metabolites were analyzed by GC-MS as described in [25]. In short, a liquid-liquid extraction was performed using chloroform:methanol:water. The quenching step using ice cold methanol was performed at the respective oxygen levels. An aliquot of the polar phase was dried, dissolved in pyridine containing methoxyamine hydrochloride and trimethylsilylated using *N*-methyl-*N*-(trimethylsilyl) trifluoroacetamide (MSTFA).

### GC-MS data processing and determination of isotopic enrichment

Deconvolution of mass spectra and targeted MID analysis were performed using MetaboliteDetector version 2.820150209R [26]. The following MetaboliteDetector peak picking and deconvolution settings were used: minimum number of peaks: 25; peak threshold: 5; minimum peak height: 5; bins/scan: 10; required base peak intensity: 0; Deconvolution width: 3 scans. An even-numbered *n*-alkane mixture (C_10_–C_40_) was measured for retention index calibration.

Non-targeted detection of stable isotope labeling and mass isotopolome analysis were performed using an in-house software based on the NTFD algorithm [13, 14, 27]. For data analysis we considered all compounds for which the NTFD algorithm detected at least under one experimental condition two mass spectrometric fragments with a coefficient of MID determination of *R*^2^>0.98, $\sum |M_{i}| < 1.05$, M_0_ abundance of 0.45<M_0_<1 and minimal enrichment 1−M_0_≥0.05. These parameters are described in more detail in [27]. Because in non-targeted analysis the maximal length of the MID vector is unknown, trailing mass isotopomer abundances of below 0.01 have been considered as noise and been removed. Compounds were identified based on RI and mass spectrum matching against an in-house reference library. Known contaminants like siloxanes were excluded from further analysis.

### Mass isotopomer abundance variation

To detect compounds with most varying labeling patterns across different experimental conditions, we analyzed the maximal standard deviation in relative mass isotopomer abundance for every compound that was detected in at least three out of five conditions:

$${} \text{variation score} = \max \sigma_{j} \quad \mid \quad \sigma_{j} = \sqrt{\frac{1}{n} \cdot \sum_{i=0}^{n} (\overline{p}_{j} - p_{j,i})^{2}} $$

where *p*_*i*,*j*_ is the relative abundance of the M_*j*_ isotopologue of the given compound in the *i*-th dataset and $\overline {p}_{j}$ the average M_*j*_ abundance across all *n* datasets. The MIDs of the heaviest common fragments across all conditions were used and the MIDs of unlabeled compounds were not considered. Compounds with the top five MID variation scores after [1,2-^13^C_2_]glucose and [U-^13^C]glutamine labeling are shown in Fig. 3a (only one of two glutamate TMS derivatives is shown).

### MID distance calculation

**Fig. 1:**
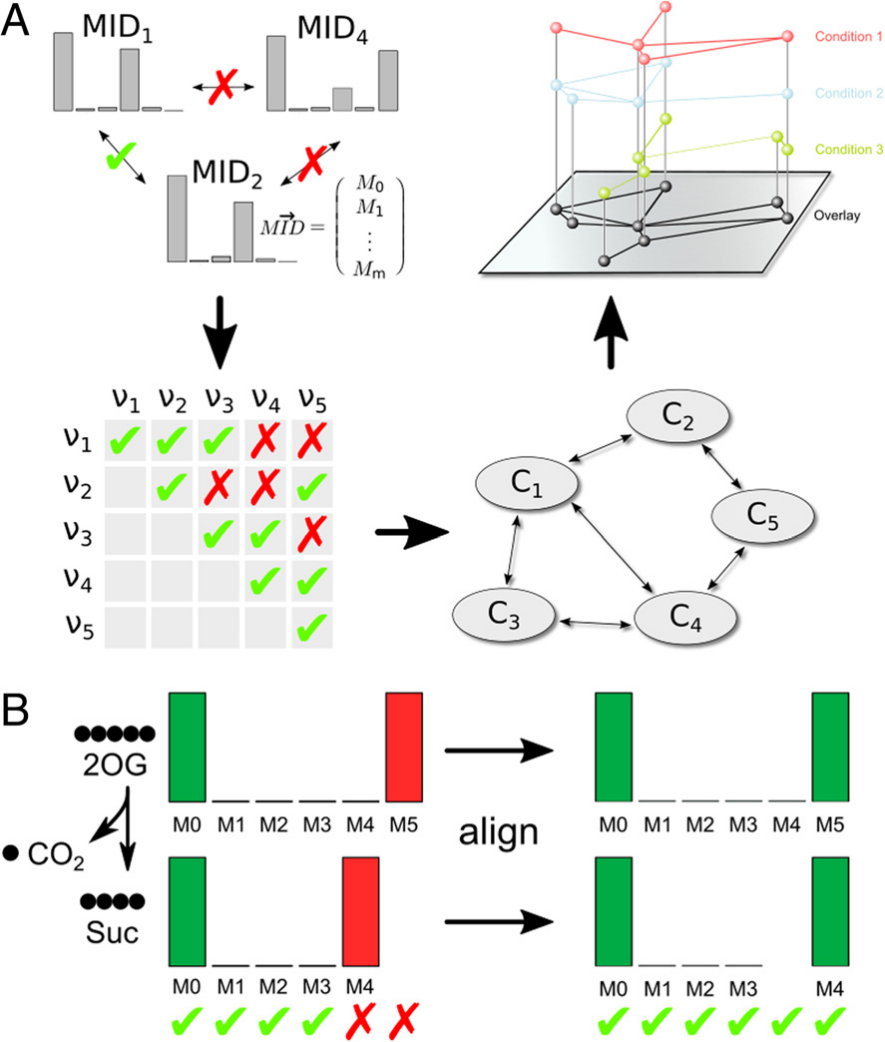
MID similarity analysis for pathway contextualization and detection of metabolically related compounds. **a** The pairwise similarities of all MIDs is determined. A similarity threshold is applied, and compounds with highly similar MIDs are visualized as network. Networks derived from different experimental conditions can be overlaid for more information. **b** Before the distance calculation the MID vectors are aligned to account for gains or losses of labeled fragments, which would otherwise conceal the metabolic proximity of these compounds

For calculation of MID distances, MIDs of the largest fragment with *R*^2^>0.95 of each compound was used. Correction for natural isotope abundance was performed using the NTFD algorithm [27]. A Needleman-Wunsch alignment was performed on the MID vectors minimizing the absolute differences in relative mass isotopomer abundances using a gap penalty of 0.4 (Fig. 1b). Subsequently, the pairwise distances of all aligned MID vectors were determined (Fig. 1a). Therefore, the Canberra distance of two MID vectors *A* and *B* was calculated as $d_{A,B} = \sum _{i=1}^{n} \frac {|A_{i}-B_{i}|}{|A_{i}|+|B_{i}|}$ and normalized by the sum of the dimensions of the MID vectors $\left (d^{norm}_{A,B} = \frac {d_{A,B}}{\text {dim} A + \text {dim}B}\right)$. The most similar compounds are shown in Fig. 4. In case of multiple TMS derivatives of the same metabolite only one is shown.

### MID deconvolution of aspartyl and acetyl moiety of *N*-acetylaspartate

The mass spectrometric fragment ions *m*/*z* 304 and *m*/*z* 245 of NAA 2TMS represent the [M-CH_3_] ^+^ and [M-CH_3_-CH_3_CONH_2_] ^+^ fragments, respectively (Additional file 1: Figure S5). The two fragments differ by the acetamido-group (CH_3_CONH_2_). The MID of NAA (given by [M-CH_3_] ^+^) is the convolution or Cauchy product [12] of the MIDs of the aspartyl moiety (M_Asp_, given by [M-CH_3_-CH_3_CONH_2_] ^+^) and the acetamido moiety (M_Ac_) of the molecule:

$$\left(\begin{array}{l} M_{0,\text{NAA}}\\ M_{1,\text{NAA}}\\ M_{2,\text{NAA}}\\ M_{3,\text{NAA}}\\ M_{4,\text{NAA}}\\ M_{5,\text{NAA}}\\ M_{6,\text{NAA}}\\ \end{array} \right) = \left(\begin{array}{ccc} M_{0,\text{Asp}} & 0 & 0\\ M_{1,\text{Asp}} & M_{0,\text{Asp}} & 0\\ M_{2,\text{Asp}} & M_{1,\text{Asp}} & M_{0,\text{Asp}}\\ M_{3,\text{Asp}} & M_{2,\text{Asp}} & M_{1,\text{Asp}}\\ M_{4,\text{Asp}} & M_{3,\text{Asp}} & M_{2,\text{Asp}}\\ 0 & M_{4,\text{Asp}} & M_{3,\text{Asp}}\\ 0 & 0 & M_{4,\text{Asp}}\\ \end{array} \right) \cdot \left(\begin{array}{c} M_{0,\text{Ac}}\\ M_{1,\text{Ac}}\\ M_{2,\text{Ac}}\\ \end{array} \right) $$

This equation system was populated with the raw mass spectral intensities and solved for M_Ac_ using a weighted least squares approach using the nlsLM routine of the minpack.lm package (version 1.1–8) for the R statistics environment (version 3.1.2). The determined acetyl MIDs were corrected for natural isotope abundance.

### siRNA transfection

For knockdown experiments, siRNA were reverse transfected into A549 cells using Lipofectamine RNAiMAX (Invitrogen/Life Technologies). For each well (12-well plate), 20 pmol siRNA were diluted in 200 *μ*l Opti-MEM I reduced-serum medium (Invitrogen/Life Technologies), supplemented with 2.5 *μ*l Lipofectamine RNAiMAX, gently mixed, and incubated for 20 min at room temperature. The prepared solution was spread in a well 5 min before 100 000 cells in 800 *μ*l of DMEM 5796 growth medium containing 10 % FBS were added. The plate was gently mixed and incubated (37 °C, 5 % CO_2_) for up to 72 h. ON-TARGETplus non-targeting and NAT8L-targeting siRNA were obtained from Dharmacon/GEHealthcare (see Additional file 1: Table S6 for target sequences).

### Growth assay

For growth assays with A549 cells, cells were transfected and cultivated at 2 % oxygen as described above. After 72 h, cells were detached using trypsin, and cell numbers and viability were determined using a Vi-CELL XR Cell Viability Analyzer (Beckman Coulter). Cell viability was above 95 % in all samples. Cell numbers are presented as mean of three independent experiments, each consisting of three wells per condition (Fig. 5).

Human bronchial epithelial BEAS-2B cells (ATCC CRL-9609), human hepatocellular carcinoma JHH-4 cells (JCRB Cell Bank) and non-neoplastic hepatocyte PH5CH8 cells [28] were transfected with non-targeting or *NAT8L*-targeting siRNA as described above and cultivated at normoxia. Cells were seeded in 12-well plates at densities of 95 000 cell/well for PH5CH8, 57 000 cells/well for JHH-4 and 100 000 cells/well for BEAS-2B. After 24 h, transfection medium was replaced by DMEM 5796 growth medium containing 10 % FBS (JHH-4 and PH5CH8) or LHC-9 medium (BEAS-2B). LHC-9 medium was prepared from LHC-8 media (Gibco) by adding 33 nM retinoic acid (Lonza) and 2.75 M epinephrine (Lonza). After 72 h, cell numbers were determined as described above. Cell numbers are presented as mean of three independent experiments, each consisting of three wells per condition.

### Metabolome analysis

For semi-quantification of metabolite levels after *NAT8L* silencing (Fig. 5), cells were incubated at 21 % O_2_ for two days after transfection. Metabolites were extracted and analyzed by GC-MS as described above. Data was analyzed using MetaboliteDetector with settings as described above, but a deconvolution width of 5 scans. Non-targeted batch quantification was performed over all data files and mass spectrometric intensities were normalized to summed analyte signal of each sample after exclusion of known contaminants. Replicates represent metabolite extracts from different cell populations (*n*=3).

## Results

### Mass isotopolome analysis

The starting point of our workflow is a stable isotope labeling experiment. After mass spectrometric measurements of labeled and unlabeled metabolite extracts, MIDs, corrected for natural isotope abundance, can be obtained in a non-targeted manner [15, 16, 27]. We analyze these metabolome-wide MIDs, the mass isotopolome, to detect changes in metabolic fluxes and exploit MID similarity between compounds for their pathway contextualization.

#### Locating flux changes by non-targeted mass isotopomer abundance variation analysis

Since changes in MIDs can only be a consequence of altered metabolic fluxes, we can reveal metabolic flux changes by detecting changes in the mass isotopolome [29]. Therefore, MIDs of identical compounds are matched across different experimental conditions to detect differences in relative mass isotopomer abundances. As a measure of variation, for each isotopically enriched compound, we calculate the maximal standard deviation of relative mass isotopomer abundance across the different experimental conditions (see Experimental Procedures). We assume that large flux changes will lead to large changes in mass isotopomer abundances, although this might neglect flux changes that make a small relative contribution to a given metabolite pool. Thus, to find the most significant flux changes, we rank metabolites by their aforementioned variation score. Like any MID analysis, this approach is limited by the facts that (1) MIDs alone can only provide relative flux information (flux ratios), and therefore, (2) not all changes in metabolic fluxes manifest in MID changes. Furthermore, as in conventional metabolomics approaches, metabolite pools of subcellular compartments are usually mixed during metabolite extraction which might reduce their informative value. Apart from that, this systematic analysis of relative mass isotopomer abundance variation detects flux changes without the requirement of any biochemical a priori knowledge on the system of interest or the identification of the respective compounds. It is only biased by analytical restrictions and the choice of the isotopic tracer and will consider any unanticipated reaction or metabolite which cannot be accounted for by current flux analysis techniques.

#### MID-similarity assisted compound identification

For subsequent interpretation of the detected changes in mass isotopomer abundances, the respective compounds need to be identified. This is usually achieved by matching their mass spectra against reference libraries [30], but the available libraries are far from comprehensive. Although thousands of chromatographic/mass spectrometric features, and among them at least several hundreds of metabolites can be analytically detected [20, 31, 32], only a fraction thereof can be identified, rendering compound identification a major bottleneck in current metabolomics research [33]. When the detected features or at least their pathways or compound classes are identified, they can provide more biological insights in addition to their function as biomarkers. Hence, compound identification is, however cumbersome, still highly important.

For compounds that are not present in reference libraries, other means for identification are required. Here, we present an approach based on MID-similarity. As described above, the MID of any metabolite is determined by those of its precursors and the flux ratio of the producing reactions. Within linear pathways, the MIDs of all compounds are identical, except if there are gains or losses of isotopically enriched fragments of the molecules. Here, we exploit the reverse conclusion, assuming that compounds with identical or highly similar MIDs are more likely to be intermediates of the same pathway or lie in the same area of the metabolic network. Therefore, by analyzing the MID similarity of different compounds, they can be grouped to metabolic pathways. Strictly speaking, this high MID similarity is only granted in linear pathways. However, in converging pathways, if one flux is much larger than the other, or there is only a dilution with the unlabeled isotopologue, then the labeling pattern of the dominating precursor is mostly conserved in the product MID and the reaction sequence can be seen as pseudo-linear. In this case, the MID similarity is still significant. Empirically, this is the case for many metabolic reactions.

To analyze MID similarity for pathway contextualization of unidentified compounds, we pairwisely compare MIDs of all isotopically enriched compounds (Fig. 1a). To account for potential losses or additions of isotopically enriched fragments to the molecules which would shift the MIDs, we perform a Needleman-Wunsch alignment [34] on the MID vectors prior to the similarity calculation (Fig. 1b). As a similarity measure, we compare the Canberra distances of all pairwisely aligned MIDs. This pairwise comparison results in a distance or similarity matrix. After applying an empirically determined distance cutoff, we create a network of compounds with higher MID similarity. The resulting graph is likely to show metabolically connected compounds. However, the MID similarity can—dependent on tracer and pathways—be ambiguous. The specificity can be increased by using distinct tracers and multiple experimental conditions (Fig. 1a). Edges in the graph occurring in multiple conditions are more likely to be biologically meaningful.

In summary, MID similarity between compounds can indicate proximity within the metabolic network. This can be used to associate unidentified compounds with identified ones, and to map them to specific pathways. This itself is valuable information and can furthermore be a strong hint for subsequent compound identification. For both identified and unidentified compounds such an MID similarity analysis can reveal new biosynthetic pathways or help to distinguish between different known ones.

#### Method summary

**Fig. 2:**
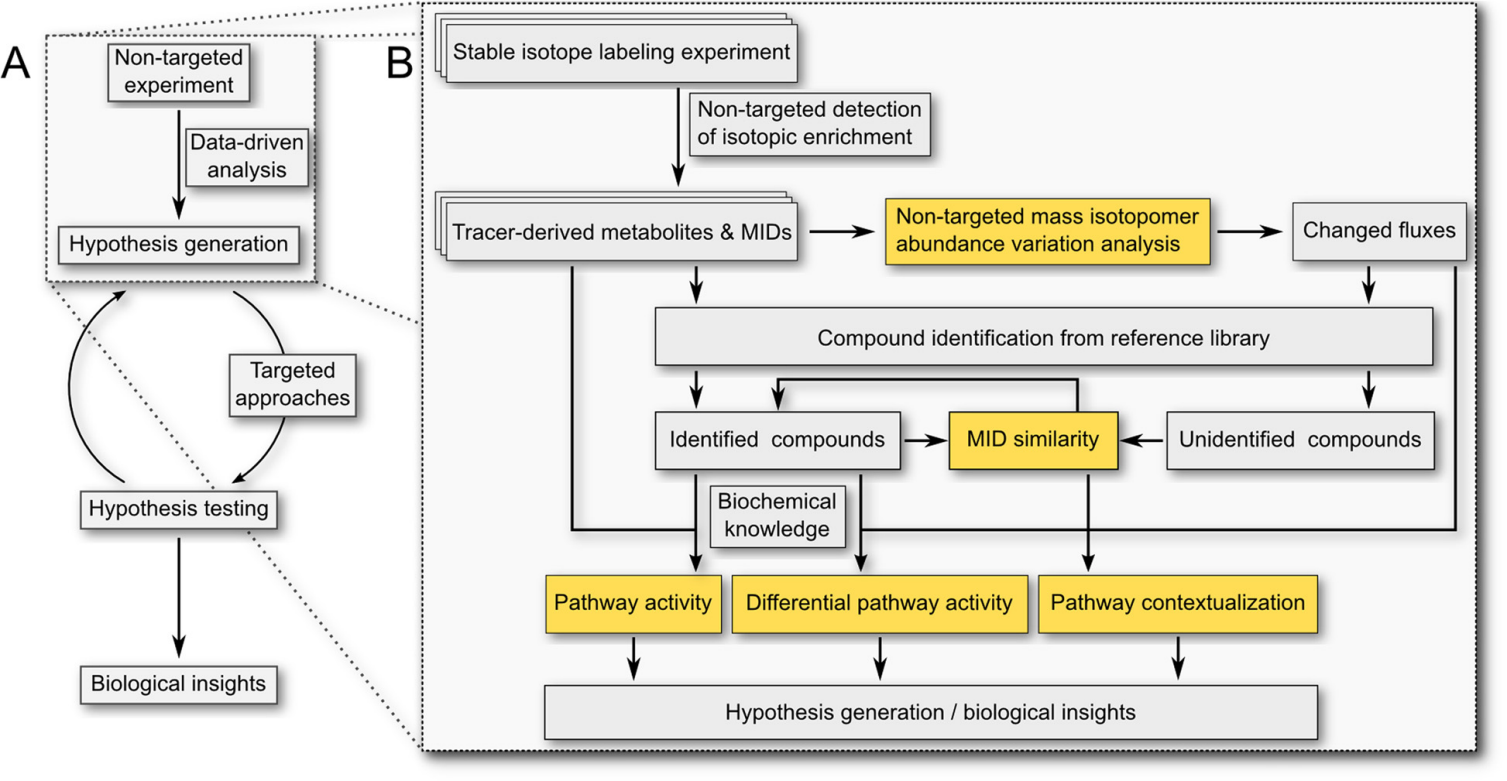
Workflow for non-targeted mass isotopolome analysis. **a** Non-targeted approaches are valuable for hypothesis generation. These hypotheses are subsequently tested by more targeted techniques leading to refined hypotheses and biological insights. **b** Data-driven analysis of non-targeted stable isotope labeling data. After stable isotope labeling experiments active pathways and changed fluxes can be detected in a non-targeted manner from metabolome-wide MIDs and changes therein. Compound identity and additional biochemical knowledge is only needed for further interpretation. Analysis of MID similarity between compounds can aid their identification or help to determine their biosynthetic pathways. *Yellow boxes* highlight new analysis techniques presented in this article

The proposed workflow starts with stable isotope labeling experiments, mass spectrometric analysis, and the non-targeted detection of isotopically enriched compounds (Fig. 2b). In addition to MIDs, such an analysis yields, for each compound, the labeled and unlabeled mass spectra, as well as the chromatographic retention time, often normalized as retention index (RI). Qualitative analysis of isotopic enrichment provides information on active fluxes and the general fate of the metabolic tracer. MIDs from different experimental conditions are systematically analyzed to detect changes in metabolic fluxes. MID similarity may indicate metabolic proximity; hence, MIDs of compounds of interest are compared to all other MIDs for pathway contextualization, discovery of potential precursors, or to facilitate identification of unidentified compounds.

Overall, this non-targeted approach provides information on (1) active pathways, (2) changed fluxes, and (3) compound identities. This information holds biological insights itself and will furthermore generate hypotheses for subsequent analyses (Fig. 2a).

An additional advantage of non-targeted isotope labeling analysis is that, depending on the proper tracer choice, it clearly shows whether a given compound is formed by the organism or was externally introduced as ingredient of an undefined growth medium or as contamination and thus provides an additional quality control. Furthermore, an advantage of the analysis of MIDs over metabolite levels is that they are more robust to technical variation than metabolite levels.

### Mass isotopolome analysis in hypoxic cancer cells

We illustrate the developed approach by analyzing human lung cancer cell metabolism under different oxygen levels. We have chosen this condition, because hypoxia induces strong changes in cellular metabolism which have been studied intensively in cancer cells [35–39]. The large number of previous studies would allow us to readily confirm our findings and to validate our approach. Furthermore, it was interesting to see if our approach was able to identify novels features. To this end, we performed stable isotope labeling experiments with [1,2-^13^C_2_]glucose and [U-^13^C]glutamine under oxygen levels ranging from 1 % O_2_ to atmospheric 21 % O_2_. We chose glucose and glutamine as isotopic tracers because they are the major carbon sources of most mammalian cells, and therefore, lead to a good metabolome coverage of isotopic enrichment. After GC-MS analysis of the metabolite extracts, we determined all isotopically enriched compounds along with their MIDs in an automated and non-targeted manner. We detected 24 compounds which were labeled from [U-^13^C]glutamine and 60 labeled from [1,2-^13^C_2_]glucose (Additional file 1: Tables S1–S2).

#### Non-targeted flux profiling reveals changes in intermediary metabolism

**Fig. 3:**
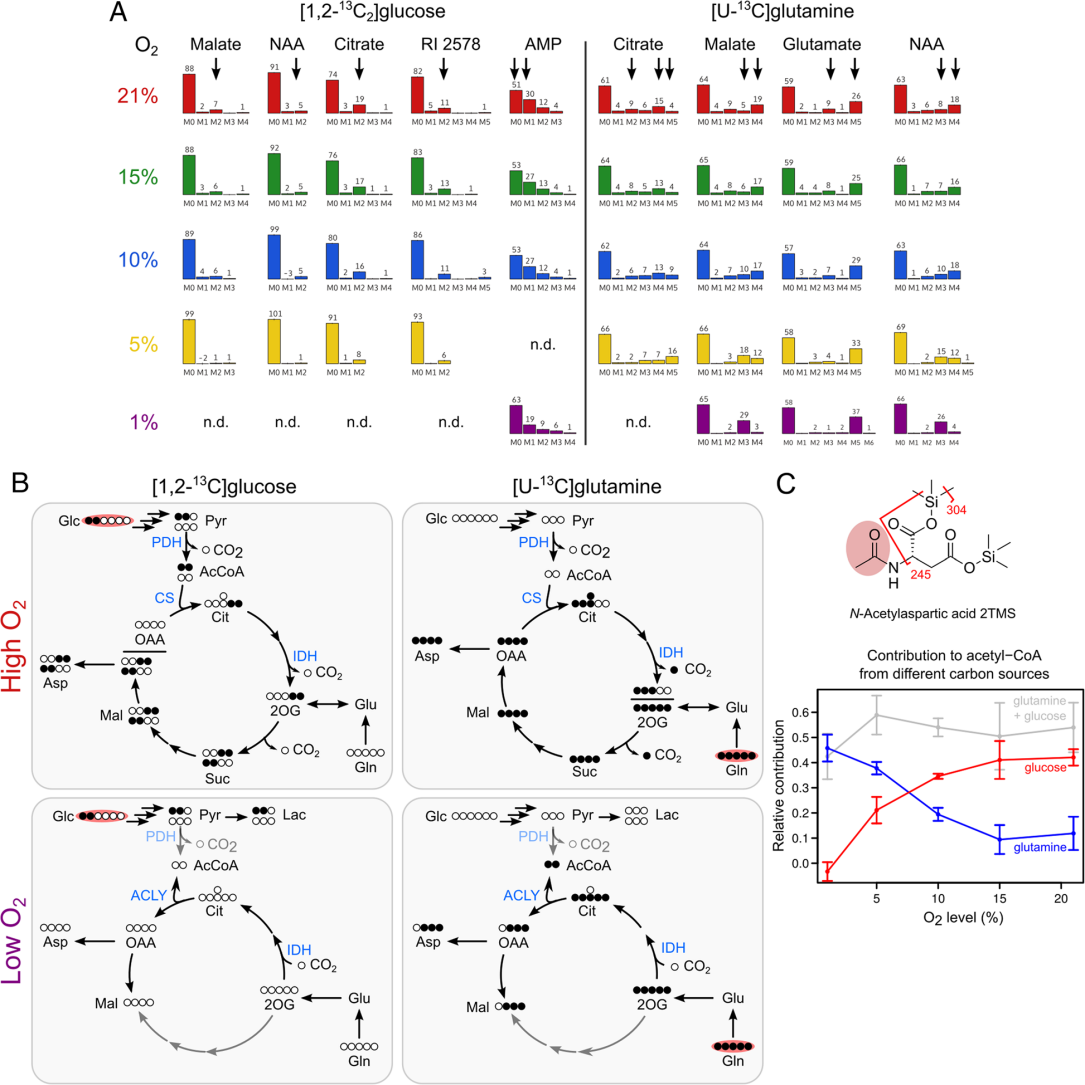
Effects of different oxygen levels on MIDs and metabolic fluxes in lung cancer cells fed with [1,2-^13^C_2_]glucose or [U-^13^C]glutamine. In total, 73 compounds were detected as isotopically enriched under at least one experimental condition. **a** Compounds with the highest variation in relative mass isotopomer abundances. Non-targeted analysis revealed that the MIDs of citric acid cycle-associated metabolites and an unidentified compound were most affected by changes in oxygen levels. *Arrows* indicate the mass isotopomers abundances with the highest variation. Unidentified compounds are named by their chromatographic retention index (RI). n.d.: not determinable. See also Additional file 1: Table S3. **b** Simplified model of how these strong changes in MIDs at low oxygen can be explained by the relative reduction of PDH flux and inversion of IDH flux directionality (see text). **c** NAA 2TMS can be used as a proxy to determine acetyl-CoA labeling under isotopic steady state conditions. Mass spectrometric fragmentation of NAA 2TMS allows to deconvolute the MIDs of the acetyl- and aspartyl-moiety (see also Additional file 1: Figure S5). Isotopic enrichment of the acetyl-moiety reflects changes in carbon origin of the acetyl-CoA pool from which NAA was synthesized. Contribution from [U-^13^C]glutamine (*blue*) or [1,2-^13^C_2_]glucose (*red*) as well as their summed contribution (*grey*) across different oxygen levels are shown. Enrichment was corrected for the applied tracer ratio and partial labeling in the case of [1,2-^13^C_2_]glucose. Data are represented as mean ±SD (3≤*n*≤4). Abbreviations: *IDH/PDH* isocitrate/pyruvate dehydrogenase, *ACLY* ATP-dependent citrate lyase, *CS* citrate synthase, *Glc* glucose, *Pyr* pyruvate, *AcCoA* acetyl-coenzyme A, *Cit* citrate, *OAA* oxaloacetate, *Mal* malate, *NAA* *N*-acetylaspartate

To detect hypoxia-induced metabolic flux changes in a non-targeted manner, we applied the aforementioned mass isotopomer abundance variation analysis and focused on the five compounds with the highest variation resulting from [U-^13^C]glutamine and [1,2-^13^C_2_]glucose labeling. Three compounds were common to both datasets, two were unique to one set (Fig. 3a, only one out of two glutamate TMS derivatives is shown).

With decreasing O_2_ levels, the compounds with high MID variation after glucose labeling showed an increase in the unlabeled (M_0_) fraction and a concomitant decrease in the abundances of heavier mass isotopomers, indicating decreased glucose contribution to their biosynthesis (Fig. 3a). The compounds with changed MIDs after glutamine labeling had an either relatively constant or slightly increasing enrichment. Additionally, three of these compounds showed a switch of the most abundant mass isotopomer indicating a change of their biosynthesis route (Fig. 3a).

While the detection of these changes in labeling patterns was fully non-targeted and did not require the compounds to be identified, the further interpretation requires their identification, as well as detailed knowledge on the metabolic network, including carbon atom transitions. Hence, to interpret the observed changes in isotopic labeling, we tried to identify the corresponding compounds by matching their mass spectra against an in-house reference library. We identified the highest-ranking metabolites from glutamine labeling as the trimethylsilyl (TMS) derivatives of malate, glutamate, citrate, and *N*-acetylaspartate (NAA) (Fig. 3a). From glucose labeling, we identified citrate and malate, NAA, and adenosine monophosphate (AMP). One compound remained unidentified; its mass spectrum could not be found in any of the common mass spectrum reference libraries such as the Golm Metabolome Database (GMD) or the NIST mass spectral library.

Citrate, malate, glutamate and NAA are all associated with the citric acid cycle. With decreasing O_2_ levels, their isotopic enrichment from [U-^13^C]glutamine changes significantly due to increasing reductive carboxylation of 2-oxoglutarate by isocitrate dehydrogenase (IDH) [36, 37] (Fig. 3b). This reductive IDH flux increased severalfold with decreasing O_2_. At the same time, isotopic enrichment from [1,2-^13^C_2_]glucose was significantly reduced, indicating a relative decrease in glucose-carbon entering the citric acid cycle as a results of pyruvate dehydrogenase (PDH) inhibition [40].

For this mass isotopomer abundance variation analysis, we used the heaviest mass spectrometric fragment that was detected as isotopically enriched. For malate, citrate and glutamate, the selected fragment all contained the full carbon backbone of the native metabolites. However, for NAA and AMP, the MIDs represent only a substructure of the analytes, so that interpretation is only possible if the fragmentation is known. The MIDs shown for fragment *m*/*z* 169 of AMP 5TMS (Fig. 3a) most likely represent the ribose moiety of AMP [41]. These labeling changes might hint towards changed fluxes through the pentose phosphate pathway, but require further validation. The MIDs of fragment *m*/*z* 245 of NAA exhibited very high similarity to malate, suggesting that this fragment contains only the aspartate moiety, but not the acetyl moiety of NAA (Fig. 3a). Combinatorial analysis of possible fragment formulas using FFC [42] indicated that this fragment most likely arises from loss of the acetamido-moiety of NAA which was confirmed by stable isotope labeling (Additional file 1: Figure S5). This turned out to be of interest because it allows for the deconvolution of the MIDs of the aspartyl- and acetyl-moiety of NAA and hence can be used as a proxy to assess acetyl-CoA labeling under isotopic steady state conditions (Fig. 3c). The determined isotopic enrichment of the NAA acetyl-moiety provides additional evidence for progressive increase of carboxylation of 2-oxoglutarate to provide acetyl-CoA for fatty acid biosynthesis under hypoxia [36].

#### MID-similarity assisted compound identification

Among the compounds with highly varying MIDs, as well as among the isotopically enriched compounds in general, there were several compounds which we were not able to identify using our in-house or any other commonly available reference library. However, to be able to interpret the observed MID changes, knowledge on their metabolic origin is imperative.

**Fig. 4:**
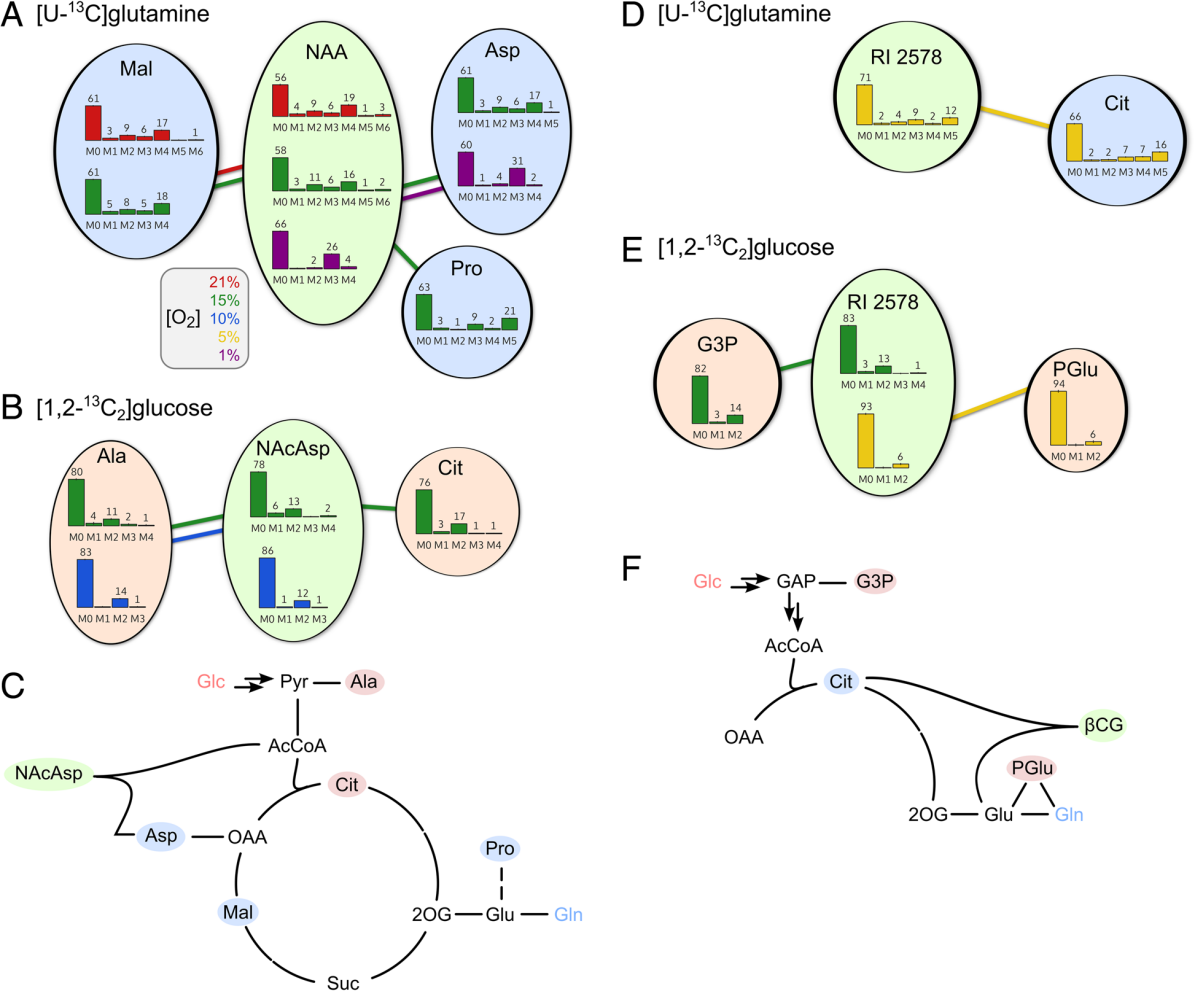
MID similarity reflects metabolic similarity and can aid compound identification. Application to an identified and an unidentified compound. **a**, **b** The networks show the compounds with the closest MIDs to *N*-acetylaspartate (NAA) after **a** [U-^13^C]glutamine or **b** [1,2-^13^C_2_]glucose labeling. *Edge color* represents the experimental condition at which the high MID similarity was observed. **c** Scheme of glycolysis and citric acid cycle showing those compounds with most similar MIDs to NAA after [U-^13^C]glutamine (*blue*) or [1,2-^13^C_2_]glucose labeling (*red*). **d**, **e** Compounds with the closest MIDs to the unidentified compound RI 2578 after **d** [U-^13^C]glutamine or **e** [1,2-^13^C_2_]glucose labeling. **f** Scheme of metabolic network showing those compounds with most similar MIDs to RI 2578 after [U-^13^C]glutamine (*blue*) or [1,2-^13^C_2_]glucose labeling (*red*), supporting its tentative identification as *β*-citrylglutamate (*β*-CG). *Mal* malate, *Asp* aspartate, *Cit* citrate, *Lac* lactate, *Glu* glutamate, *Gln* glutamine, *Suc* succinate, *OAA* oxaloacetate, *2OG* 2-oxoglutarate, *Pro* proline. See also Additional file 1: Table S4

To show that the aforementioned MID similarity analysis can be a valuable tool to aid compound identification, we first performed MID similarity analysis on the already identified NAA. The compounds with the most similar labeling patterns after [U-^13^C]glutamine labeling were aspartate, malate and pyroglutamate (Fig. 4a), and alanine and citrate after [1,2-^13^C_2_]glucose labeling (Fig. 4b). Aspartate is the direct precursor of NAA and the other compounds are only a few reactions away, demonstrating that MID similarity analysis can indeed provide valuable hints on metabolically closely related compounds (Fig. 4c).

Next, we performed the same analysis to aid identification of the yet unidentified compound RI 2578 which popped up in the MID variation analysis (Fig. 3a). The heaviest fragment that was detected as isotopically enriched showed very high MID similarity to citrate after [U-^13^C]glutamine labeling, suggesting that the unidentified compounds must be closely related to citrate (Fig. 4d). The compounds with high MID similarity after [1,2-^13^C_2_]glucose labeling, glycerol 3-phosphate and pyroglutamate, are very distantly related, and therefore, are less informative (Fig. 4e). The analyzed fragment *m*/*z* 363 has only about half the mass of the heaviest fragment *m*/*z* 666 of the mass spectrum (Additional file 1: Figure S7). For the heaviest fragment, no enrichment patterns could be determined due to its low abundance and low enrichment. We assumed the heaviest fragment *m*/*z* 666 was the [M-CH_3_] ^+^ ion, a fragment ion that is commonly observed after electron ionization of TMS derivatives in place of the molecular ion [M] ^+^. In order to identify compound RI 2578, we searched the HMDB database [43] for the mass of the native metabolite, assuming due to its retention index 5 or 6 TMS groups in the molecule. Of the 38 database hits, *β*-citrylglutamate (*β*-CG) was the candidate that could account for the observed high MID similarity to citrate (Fig. 4d, f). Unfortunately, there was neither an authentic standard of *β*-CG commercially available to confirm this tentative identification, nor was there an electron ionization mass spectrum published. However, as discussed below, there is biological evidence supporting this identification.

### NAT8L-mediated NAA biosynthesis in lung cancer cells

We were intrigued by finding NAA in lung cancer cells. Its isotopic enrichment clearly indicated its de novo biosynthesis there, although so far biosynthesis was assumed to be restricted to neuronal tissue. Therefore, we followed up on this finding to investigate whether NAA plays a significant role in cancer cells. The recent study by Lou et al. [23], reporting some of the results presented below, was only published during the preparation of this manuscript.

**Fig. 5:**
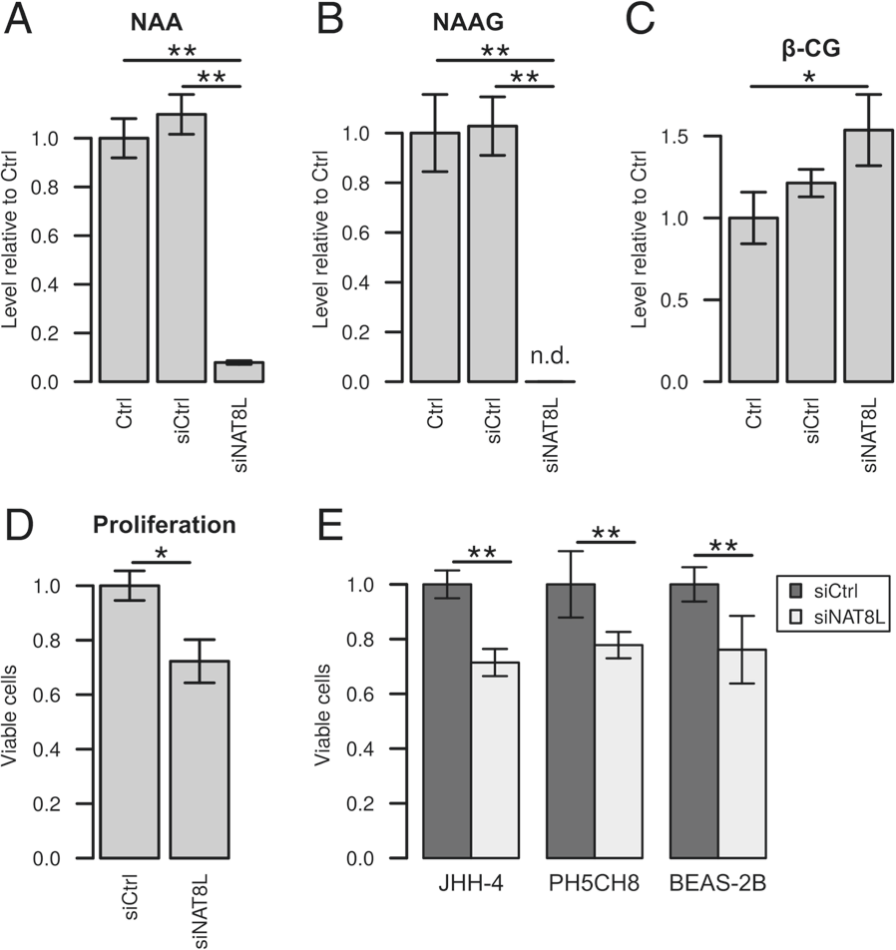
Effects of siRNA-mediated silencing of *NAT8L*. Transfection of A549 cells with *NAT8L* targeting siRNA (siNAT8L) leads to a significant reduction of **a** NAA and **b** NAAG levels, and **c** an increase in *β*-CG as compared to transfection with a non-targeting siRNA (siCtrl) or untreated cells (Ctrl). NAAG levels after *NAT8L* silencing were below the detection limit (n.d.: not determinable). **d** Proliferation of A549 cells is significantly reduced upon transfection with *NAT8L* targeting siRNA (siNAT8L) as compared to transfection with a non-targeting siRNA (siCtrl). Cell numbers 72 h after siRNA transfection are normalized to average of siCtrl. **e** Proliferation of hepatocellular carcinoma cells (JHH-4), bronchial epithelial cells (BEAS-2B), and non-neoplastic hepatocytes (PH5CH8) is significantly reduced upon transfection with *NAT8L* targeting siRNA (siNAT8L) as compared to transfection with a non-targeting siRNA (siCtrl). Cell numbers 72 h after siRNA transfection are normalized to average of siCtrl. Data are presented as means ± standard deviation of *n*=3 replicates (**a**–**c**) or three independent experiments with three technical replicates (*n* = 9); *asterisks* indicate Welsh’s *t* test *p* values of below 0.05 (*) or 0.01 (**)

In neurons, NAA is known to be produced by NAT8L [44]. To confirm NAT8L as the producing enzyme in A549 cells, we transfected them with *NAT8L* targeting siRNA and analyzed polar metabolite extracts by GC-MS. Upon *NAT8L* silencing, NAA levels were drastically reduced (Fig. 5a), confirming NAT8L-mediated biosynthesis of NAA. Besides, NAA, we also observed production of another neuropeptide, *N*-acetylaspartylglutamate (NAAG), by A549 cells. NAAG is synthesized from NAA by RIMKLA and RIMKLB, the latter of which also catalyzes the formation of *β*-CG as alternative product [45]. Both *RIMKLA* and *RIMKLB* were found to be expressed in A549 as determined by qPCR analysis (data not shown). Upon transfection with *NAT8L* targeting siRNA, NAAG levels dropped below the detection limit (Fig. 5b), whereas the levels of putative *β*-CG increased (Fig. 5c). As mentioned above, there was unfortunately no authentic standard available to confirm the identity of RI 2578 as *β*-CG. Yet, its identification is corroborated by mass spectrometric fragmentation (Additional file 1: Figure S7), stable isotope labeling data as well as by the correlation of *NAT8L* silencing with an increase in levels of RI 2578.

#### Loss of NAT8L function impairs cell proliferation

Others have observed a positive correlation between NAA levels and malignancy of prostate tumors [46] and identified NAA as a potential diagnostic blood biomarker for cancer [23]. To determine whether NAA or NAT8L has an effect on cell proliferation, we performed growth assays after siRNA-mediated *NAT8L* silencing. After 72 h, the number of cells transfected with NAT8L-targeting siRNA was 28 % lower than the ones transfected with a non-targeting siRNA, suggesting a substantial role of NAA or NAT8L in A549 cells (Fig. 5d). To confirm this finding, we repeated the experiments using bronchial epithelial BEAS-2B cells, hepatocellular carcinoma JHH-4 cells, and non-neoplastic hepatocyte PH5CH8 cells. All three cell lines showed a similar reduction in cell numbers of about 20–30 % after transfection with *NAT8L* targeting siRNA (Fig. 5e).

## Discussion

Non-targeted data acquisition and analysis approaches are valuable tools to generate initial hypotheses, especially when little a priori information is available on the organism or subject of interest. We showed how stable isotope labeling experiments and subsequent non-targeted mass isotopolome analysis can be used for metabolic flux profiling and hypothesis generation (Fig. 2a).

To demonstrate our novel non-targeted stable isotope labeling analysis workflow, we performed stable isotope labeling experiments with human adenocarcinoma cells incubated in the presence of ^13^C-labeled glucose and glutamine at various oxygen levels and analyzed the resulting mass isotopolome. We illustrated that our approach can be used to extract biologically meaningful information instead of mere statistical differences. Although we applied our workflow on GC-MS data, it can also directly be used in LC-MS experiments which may provide higher metabolome coverage.

We globally analyzed mass isotopomer abundance variation across different experimental conditions to detect metabolic flux changes in a non-targeted manner. MIDs after stable isotope labeling are determined by metabolic fluxes. This relationship is already exploited in ^13^C-MFA, but only in a highly targeted manner. Here, we applied a global differential MID analysis to detect metabolic flux changes without considering the identity of the respective compounds. Therefore, even if such flux changes occurred in yet unknown reactions, they would pop up in this analysis, rendering compound identification dispensable at this stage of the analysis. Since for many organisms or cell types the metabolic network is not fully known, such a non-targeted and data-driven approach is highly desirable. It will not replace subsequent targeted experiments, but it can be a valuable scouting strategy to not miss unanticipated reactions or metabolites and to validate the assumptions required for example for ^13^C-MFA.

To demonstrate and validate our approach, we analyzed cellular metabolism of lung cancer cells at different oxygen levels. The data-driven analysis of isotopic enrichment correctly identified enhanced reductive carboxylation of 2-oxoglutarate to isocitrate by IDH and subsequent cleavage of citrate by ACLY to produce cytosolic acetyl-CoA which flows into increased fatty acid biosynthesis [36–38] (Fig. 3). This inversion of IDH flux directionality is an important feature of cancer cells since it allows for the generation of acetyl-CoA from glutamine under hypoxia when acetyl-CoA production from glucose is strongly inhibited. The non-targeted detection of these previously reported findings validate our approach.

We argued that, because MID similarity often correlates with metabolic proximity, comparison of MIDs of different compounds can reveal metabolic similarity. Addressing a current bottleneck in non-targeted metabolomics studies, we demonstrated how the similarity in MIDs after stable isotope labeling can facilitate compound identification. Knowledge of biochemically related compounds helps to constrain database searches and it can help to elucidate unknown biosynthetic pathways by revealing potential precursors. Although MID similarity analysis will not always allow for compound identification, it can still be used to put unknown compounds into the context of certain biochemical pathways or to provide hypotheses on chemical substructures. MID similarity can, dependent on tracer and pathways, sometimes be ambiguous. However, this ambiguity can be reduced by the use of distinct tracers and multiple experimental conditions as done in this study. Analysis of MID similarity can additionally provide valuable hints on the composition of a given mass spectrometric fragment, as shown above for NAA.

A related approach to our MID similarity analysis is the recently described “metabolic turnover analysis” [47] which analyzes correlations in mass isotopomer abundances to determine metabolic distances. Whereas Nakayama et al. [47] analyzed time-series data from a transient labeling experiment, we compare MIDs from only one single time-point. The dynamic nature of metabolic turnover analysis has the advantage, that it can reveal the sequence of intermediates of linear pathways, an information that is lost in isotopic steady state. However, this extra information comes at the cost of a more complex experimental setup.

We demonstrated the value of our MID similarity analysis in the case of the already identified NAA and an unidentified compound, the spectrum of which was not present in any of the common reference libraries (Fig. 4). This MID similarity analysis constrained the database search for the given nominal mass to only one candidate structure. This putative identification is corroborated by mass spectrometric fragmentation, isotope labeling, and a gene silencing experiment.

Using stable isotope labeling and siRNA-mediated gene silencing, we showed that A549 lung cancer cells produce NAA and its downstream metabolite and neuropeptide NAAG (Fig. 5). NAA is highly abundant in the brain and was long thought to only be produced in neuronal tissues. Its functions are still not fully understood, but several potential roles are discussed [48, 49]. In the context of cancer, NAA has not received much attention, except for tumors of the brain where NAA concentrations are high per se. Increased levels of NAA were detected in ovarian tumors [50–54], and a positive correlation between urinary NAA levels and malignancy of prostate cancer cells has been observed [46]. Only during the preparation of this manuscript, two independent studies focusing on NAA and NAT8L were published [23, 55]. At the time of our study, it was not yet clear whether increased NAA levels in cancer cells are due to endogenous biosynthesis or accumulation of exogenous NAA. Here, using ^13^C-labeling, we showed that lung adenocarcinoma cells are able to produce this metabolite via the NAT8L enzyme. Production of NAA is not limited to A549 cells; we also detected NAA in prostate (RWPE-2) and liver (HepG2) cancer cell lines (unpublished observations) and in bronchial epithelial cells (BEAS-2B) non-neoplastic hepatocytes (PH5CH8). Lou et al. investigated NAA levels and *NAT8L* expression levels in lung cancer in parallel and independently of our studies and their findings were published during the preparation of our manuscript [23]. Here, we confirm their report on NAA production in A549 cells and show furthermore that the NAA downstream metabolite NAAG is produced and that *NAT8L* silencing inhibits cell proliferation (Fig. 5). Production of NAA and a negative effect of NAT8L silencing on proliferation has also been reported by Zand et al. in ovarian cancer cells [55]. Furthermore, they showed a lower survival rate of cancer patients with higher NAT8L and NAA levels. These data hint towards a more integral role of NAA in cancer cells, more than a mere biomarker or byproduct, which needs to be investigated further.

Nothing is known about the mechanistic role of either NAA, NAAG, or NAT8L in cancer yet. Exogenous NAA was found to increase proliferation of glioma stem-like cells [56]. Pessentheiner et al. reported that NAT8L plays an important role in lipid metabolism in brown adipocytes [57]. Its functional role there is yet unclear, but the authors hypothesized an *N*-acetylaspartate-based acetyl-CoA shuttle across the mitochondrial membrane, as suggested for neurons [58, 59] and similar to the intercellular acetyl-transport observed in the brain [48]. In this model, NAA is exported from the mitochondria, deacetylated by cytosolic aspartoacylase (ASPA), and the resulting acetate is activated by cytosolic acetyl-CoA synthase (ACSS2). Such a shuttling mechanism to provide cytosolic acetyl-CoA would also be advantageous for cancer cells which are known to have increased fatty acid biosynthesis. NAA-based acetyl-CoA shuttling would decouple acetyl-transport from citrate synthesis for oxidation in the citric acid cycle. Furthermore, it would explain recent findings on the importance of ACSS2 in cancer cells [60–62]. The inhibition of cell proliferation we observed here upon *NAT8L* silencing would be in line with NAA-mediated acetyl transport. Lack of NAT8L could induce a shortage of cytosolic acetyl-CoA for fatty acid biosynthesis and other metabolic processes which would eventually reduce cell proliferation. More data are required to unravel the exact mechanism by which NAT8L confers a growth advantage to these cells. This acetyl-shuttling relies on mitochondrial NAA biosynthesis. However, there is no consent about subcellular localization of NAT8L [63–68].

If NAT8L was only localized in the cytosol, NAA could still play an important role as precursor of NAAG, which we also detected in A549 cells. NAAG was reported as an agonist at type II metabotropic glutamate receptors [69, 70] which are also expressed by cancer cells [71] and have been shown to promote cell proliferation [72]. The observed reduction in cell proliferation after *NAT8L* silencing could therefore be due to reduced levels of NAAG.

An alternative product of RIMKLB, one of the NAAG-producing enzymes, is *β*-CG [45] which we putatively identified in A549 cells. Very little is known about the function of this metabolite. It was suggested to act as an iron carrier, able to reactivate aconitase activity and increasing cell viability [73]. It is unclear whether *β*-CG plays a specific role in A549 cells, or whether it only forms as a side product of RIMKLB.

Besides any biological role, NAA is of analytical interest because of its mass spectrometric fragmentation. Under isotopic steady state conditions, it can be used to deduce labeling of the acetyl-CoA pool from which it is synthesized (Fig. 3). Acetyl-CoA is a hub of many anabolic and catabolic reactions, linking fatty acid, carbohydrate and amino acids metabolism, and is a substrate for protein acetylation. Acetyl-CoA is not directly accessible via GC-MS measurements, but its isotopic enrichment can alternatively be deduced from labeling patterns of fatty acids by isotopomer spectral analysis (ISA) [74]. However, this requires additional sample processing and measurements and is further complicated by the fact that cellular fatty acids can be derived from medium components directly or by elongation or be synthesized de novo. When the localization of NAT8L is resolved, NAA may furthermore provide a means to analyze isotopic enrichment of a compartment-specific acetyl-CoA pool.

Analyzing isotopic enrichment of the acetyl-moiety of NAA at different oxygen levels revealed that with decreasing oxygen availability, acetyl-CoA was progressively increasingly derived from glutamine instead of glucose, whereas their combined contribution was relatively stable.

## Conclusions

We applied stable isotope labeling and illustrated a novel non-targeted mass isotopolome analysis approach to systematically analyze the metabolic hypoxia response of human lung cancer cells. We employed non-targeted mass isotopomer abundance variation analysis for non-targeted metabolic flux change profiling and showed how MID similarity can assist compound identification, addressing a major bottleneck of current metabolomics research. This approach can also account for unknown or unanticipated reactions, thus bridging the gap between non-targeted metabolomics and ^13^C-MFA. With this data-driven analysis, we detected known hypoxia-induced metabolic effects, validating our approach. Furthermore, this analysis led to the discovery of a potentially important role of NAA in cancer cell metabolism, which needs to be investigated further. In summary, this non-targeted approach provided biological insights and proved to be a fruitful methodology for hypothesis generation for subsequent more targeted analyses.

## Additional file

Additional file 1 Supplemental information. List of isotopically enriched compounds and their mass isotopomer distributions; selected mass spectra. (PDF 308 kb)
