# Supplementary material for: Bridging the gap between non-targeted stable isotope labeling and metabolic flux analysis
Source: Cancer Metab. 2016 Apr 23;4:10. doi: 10.1186/s40170-016-0150-z (PMC4842284; doi:10.1186/s40170-016-0150-z)
Supplement: Additional file 1 — Supplemental information. List of isotopically enriched compounds and their mass isotopomer distributions; selected mass spectra. (PDF 308 kb) [file 40170_2016_150_MOESM1_ESM.pdf]

# Bridging the gap between non-targeted stable isotope labeling and metabolic flux analysis

Supplemental Information

Daniel Weindl, Thekla Cordes, Xiangyi Dong, Andre Wegner, Karsten Hiller

## List of Tables and figures

**S1** List compounds detected as isotopically enriched after [1,2-$^{13}C_2$]glucose labeling. . . . . 2
**S2** List compounds detected as isotopically enriched after [U-$^{13}C$]glutamine labeling. . . . . 11
**S3** Metabolites, derivatives and ions shown in Figure 3 (MID variation) . . . 16
**S4** Metabolites, derivatives and ions shown in Figure 4 (MID similarity) . . 16
**S5** Fragmentation of *N*-acetylaspartic acid 2TMS. . . . . 17
**S6** siRNA target sequences for gene silencing . . . . . 17
**S7** Putative structure and fragmentation of RI 2578 . . . . . 18

**Table S1:** All compounds which have been detected as isotopically enriched along with their corrected MIDs of the largest detected fragment after [1,2-$^{13}C_2$]glucose.

| **Metabolite** | **RI** | **Ions (M0)** | **M** | **21% $O_2$** | **15% $O_2$** | **10% $O_2$** | **5% $O_2$** | **1% $O_2$** |
|---|---|---|---|---|---|---|---|---|
| RI 981 | 980.688 | 184 0 0 0 0 | 0 | 0.990 | *NA* | *NA* | *NA* | *NA* |
| | | | 1 | -0.018 | *NA* | *NA* | *NA* | *NA* |
| | | | 2 | 0.030 | *NA* | *NA* | *NA* | *NA* |
| Lactic acid 2TMS | 1061.92 | 219 0 219 219 219 | 0 | 0.795 | *NA* | 0.794 | 0.798 | 0.802 |
| | | | 1 | 0.024 | *NA* | 0.025 | 0.022 | 0.020 |
| | | | 2 | 0.194 | *NA* | 0.195 | 0.195 | 0.202 |
| Alanine 2TMS | 1105.03 | 190 190 190 190 190 | 0 | 0.851 | 0.850 | 0.832 | 0.819 | 0.849 |
| | | | 1 | 0.023 | 0.013 | 0.010 | 0.019 | 0.015 |
| | | | 2 | 0.144 | 0.148 | 0.150 | 0.179 | 0.143 |
| | | | 3 | *NA* | *NA* | 0.011 | *NA* | *NA* |
| Proline 1TMS | 1172.79 | 172 0 0 0 0 | 0 | 0.895 | *NA* | *NA* | *NA* | *NA* |
| | | | 1 | 0.030 | *NA* | *NA* | *NA* | *NA* |
| | | | 2 | 0.079 | *NA* | *NA* | *NA* | *NA* |
| Serine 2TMS | 1257.33 | 132 132 132 0 0 | 0 | 0.904 | 0.928 | 0.944 | *NA* | *NA* |
| | | | 1 | 0.050 | 0.048 | 0.028 | *NA* | *NA* |
| | | | 2 | 0.043 | 0.022 | 0.041 | *NA* | *NA* |
| Glycerol 3TMS | 1277.11 | 218 218 0 0 218 | 0 | 0.936 | 0.931 | *NA* | *NA* | 0.916 |
| | | | 1 | 0.015 | 0.012 | *NA* | *NA* | 0.015 |
| | | | 2 | 0.051 | 0.063 | *NA* | *NA* | 0.080 |
| Proline 2TMS | 1296.26 | 0 244 0 0 0 | 0 | *NA* | 0.909 | *NA* | *NA* | *NA* |
| | | | 1 | *NA* | 0.022 | *NA* | *NA* | *NA* |
| | | | 2 | *NA* | 0.070 | *NA* | *NA* | *NA* |
| Glycine 3TMS | 1305.68 | 0 0 0 158 0 | 0 | *NA* | *NA* | *NA* | 0.946 | *NA* |
| | | | 1 | *NA* | *NA* | *NA* | 0.031 | *NA* |
| | | | 2 | *NA* | *NA* | *NA* | 0.025 | *NA* |
| | | | 3 | *NA* | *NA* | *NA* | -0.011 | *NA* |

Continued on next page

Table S1 – *continued from previous page*

| Metabolite | RI | Ions (M0) | M | 21% $O_2$ | 15% $O_2$ | 10% $O_2$ | 5% $O_2$ | 1% $O_2$ |
|---|---|---|---|---|---|---|---|---|
| | | | 4 | *NA* | *NA* | *NA* | 0.014 | *NA* |
| RI 1313 | 1312.95 | 0 173 0 0 0 | 0 | *NA* | 0.803 | *NA* | *NA* | *NA* |
| | | | 1 | *NA* | 0.010 | *NA* | *NA* | *NA* |
| | | | 2 | *NA* | 0.194 | *NA* | *NA* | *NA* |
| Serine 3TMS | 1360.03 | 278 278 278 278 0 | 0 | 0.938 | 0.941 | 0.963 | 0.938 | *NA* |
| | | | 1 | 0.038 | 0.032 | 0.010 | 0.035 | *NA* |
| | | | 2 | 0.024 | 0.028 | 0.038 | 0.037 | *NA* |
| Aspartic acid 2TMS | 1419.6 | 160 160 0 0 0 | 0 | 0.884 | 0.883 | *NA* | *NA* | *NA* |
| | | | 1 | 0.063 | 0.057 | *NA* | *NA* | *NA* |
| | | | 2 | 0.043 | 0.055 | *NA* | *NA* | *NA* |
| | | | 3 | 0.012 | *NA* | *NA* | *NA* | *NA* |
| Malic acid 3TMS | 1487.79 | 335 335 335 335 0 | 0 | 0.882 | 0.889 | 0.894 | 0.997 | *NA* |
| | | | 1 | 0.026 | 0.034 | 0.047 | -0.021 | *NA* |
| | | | 2 | 0.078 | 0.068 | 0.060 | 0.013 | *NA* |
| | | | 3 | 0.004 | 0.000 | 0.011 | 0.017 | *NA* |
| | | | 4 | 0.017 | 0.015 | *NA* | *NA* | *NA* |
| Pyroglutamic acid 2TMS | 1516.67 | 0 0 0 214 0 | 0 | *NA* | *NA* | *NA* | 0.940 | *NA* |
| | | | 1 | *NA* | *NA* | *NA* | 0.006 | *NA* |
| | | | 2 | *NA* | *NA* | *NA* | 0.063 | *NA* |
| Aspartic acid 3TMS | 1519.17 | 334 334 334 0 0 | 0 | 0.892 | 0.879 | 0.924 | *NA* | *NA* |
| | | | 1 | 0.039 | 0.017 | 0.013 | *NA* | *NA* |
| | | | 2 | 0.062 | 0.083 | 0.059 | *NA* | *NA* |
| | | | 3 | *NA* | 0.023 | *NA* | *NA* | *NA* |
| RI 1530 | 1529.69 | 216 216 216 0 0 | 0 | 0.789 | 0.787 | 0.793 | *NA* | *NA* |
| | | | 1 | 0.016 | 0.018 | 0.018 | *NA* | *NA* |
| | | | 2 | 0.205 | 0.203 | 0.201 | *NA* | *NA* |
| Glutamic acid 2TMS | 1531.47 | 276 276 276 276 0 | 0 | 0.894 | 0.892 | 0.910 | 0.963 | *NA* |
| | | | 1 | 0.011 | 0.021 | 0.022 | 0.001 | *NA* |

Continued on next page

**Table S1 – *continued from previous page***

| Metabolite | RI | Ions (M0) | M | 21% $O_2$ | 15% $O_2$ | 10% $O_2$ | 5% $O_2$ | 1% $O_2$ |
|---|---|---|---|---|---|---|---|---|
| | | | 2 | 0.093 | 0.074 | 0.073 | 0.034 | *NA* |
| | | | 3 | *NA* | -0.003 | *NA* | *NA* | *NA* |
| | | | 4 | *NA* | 0.012 | *NA* | *NA* | *NA* |
| 2-Hydroxypent-2-endioic acid 3TMS | 1604.44 | 347 347 0 0 0 | 0 | 0.864 | 0.890 | *NA* | *NA* | *NA* |
| | | | 1 | 0.037 | 0.010 | *NA* | *NA* | *NA* |
| | | | 2 | 0.081 | 0.086 | *NA* | *NA* | *NA* |
| | | | 3 | 0.012 | 0.004 | *NA* | *NA* | *NA* |
| | | | 4 | 0.013 | 0.019 | *NA* | *NA* | *NA* |
| Glutamic acid 3TMS | 1617.07 | 348 348 348 0 0 | 0 | 0.901 | 0.899 | 0.936 | *NA* | *NA* |
| | | | 1 | 0.022 | 0.023 | 0.010 | *NA* | *NA* |
| | | | 2 | 0.085 | 0.085 | 0.060 | *NA* | *NA* |
| RI 1627 | 1627.07 | 124 124 124 0 0 | 0 | 0.877 | 0.917 | 0.918 | *NA* | *NA* |
| | | | 1 | 0.035 | 0.031 | 0.025 | *NA* | *NA* |
| | | | 2 | 0.080 | 0.069 | 0.062 | *NA* | *NA* |
| | | | 3 | 0.010 | *NA* | *NA* | *NA* | *NA* |
| | | | 4 | 0.005 | *NA* | *NA* | *NA* | *NA* |
| | | | 5 | 0.015 | *NA* | *NA* | *NA* | *NA* |
| RI 1634 | 1633.9 | 0 0 0 0 247 | 0 | *NA* | *NA* | *NA* | *NA* | 0.486 |
| | | | 1 | *NA* | *NA* | *NA* | *NA* | 0.449 |
| | | | 2 | *NA* | *NA* | *NA* | *NA* | 0.061 |
| N-acetylaspartic acid 2TMS | 1651.79 | 245 245 245 245 0 | 0 | 0.917 | 0.927 | 0.995 | 1.014 | *NA* |
| | | | 1 | 0.040 | 0.021 | -0.034 | 0.006 | *NA* |
| | | | 2 | 0.051 | 0.056 | 0.058 | 0.016 | *NA* |
| RI 1701 | 1701.11 | 218 218 0 218 218 | 0 | 0.795 | 0.795 | *NA* | 0.772 | 0.808 |
| | | | 1 | 0.014 | 0.022 | *NA* | 0.017 | 0.014 |
| | | | 2 | 0.193 | 0.190 | *NA* | 0.183 | 0.195 |
| | | | 3 | *NA* | *NA* | *NA* | 0.032 | *NA* |
| RI 1716 | 1715.91 | 307 307 307 307 307 | 0 | 0.790 | 0.830 | 0.833 | 0.795 | 0.844 |

Continued on next page

Table S1 – *continued from previous page*

| Metabolite | RI | Ions (M0) | M | 21% $O_2$ | 15% $O_2$ | 10% $O_2$ | 5% $O_2$ | 1% $O_2$ |
|---|---|---|---|---|---|---|---|---|
| | | | 1 | 0.086 | 0.055 | 0.053 | 0.114 | 0.066 |
| | | | 2 | 0.115 | 0.108 | 0.138 | 0.111 | 0.087 |
| | | | 3 | *NA* | -0.002 | *NA* | *NA* | 0.016 |
| | | | 4 | *NA* | 0.014 | *NA* | *NA* | *NA* |
| Xylitol 5TMS | 1720.45 | 319 319 319 319 319 | 0 | 0.646 | 0.674 | 0.654 | 0.631 | 0.614 |
| | | | 1 | 0.229 | 0.205 | 0.213 | 0.224 | 0.225 |
| | | | 2 | 0.110 | 0.105 | 0.109 | 0.119 | 0.129 |
| | | | 3 | 0.014 | 0.014 | 0.020 | 0.022 | 0.025 |
| | | | 4 | *NA* | *NA* | 0.013 | 0.014 | *NA* |
| RI 1734 | 1734.44 | 217 217 217 217 0 | 0 | 0.646 | 0.648 | 0.645 | 0.648 | *NA* |
| | | | 1 | 0.328 | 0.322 | 0.330 | 0.324 | *NA* |
| | | | 2 | 0.032 | 0.035 | 0.030 | 0.031 | *NA* |
| Glycerol-3-phosphate 4TMS | 1757.53 | 357 357 0 357 0 | 0 | 0.816 | 0.828 | *NA* | 0.831 | *NA* |
| | | | 1 | 0.021 | 0.030 | *NA* | 0.013 | *NA* |
| | | | 2 | 0.133 | 0.148 | *NA* | 0.140 | *NA* |
| | | | 3 | 0.017 | *NA* | *NA* | 0.025 | *NA* |
| | | | 4 | 0.025 | *NA* | *NA* | *NA* | *NA* |
| RI 1763 | 1763.39 | 217 217 0 0 0 | 0 | 0.616 | 0.606 | *NA* | *NA* | *NA* |
| | | | 1 | 0.051 | 0.058 | *NA* | *NA* | *NA* |
| | | | 2 | 0.382 | 0.376 | *NA* | *NA* | *NA* |
| RI 1774 | 1774.3 | 292 292 0 0 0 | 0 | 0.569 | 0.601 | *NA* | *NA* | *NA* |
| | | | 1 | 0.420 | 0.382 | *NA* | *NA* | *NA* |
| | | | 2 | 0.007 | 0.030 | *NA* | *NA* | *NA* |
| | | | 3 | 0.017 | *NA* | *NA* | *NA* | *NA* |
| Fructose 5TMS MP | 1796.75 | 319 319 319 319 319 | 0 | 0.938 | 0.959 | 0.962 | 0.936 | 0.963 |
| | | | 1 | 0.039 | 0.021 | 0.017 | 0.027 | 0.002 |
| | | | 2 | 0.024 | 0.022 | 0.025 | 0.031 | 0.001 |
| | | | 3 | *NA* | *NA* | *NA* | 0.017 | 0.052 |

Continued on next page

**Table S1 – *continued from previous page***

| Metabolite | RI | Ions (M0) | M | 21% $O_2$ | 15% $O_2$ | 10% $O_2$ | 5% $O_2$ | 1% $O_2$ |
|---|---|---|---|---|---|---|---|---|
| Fructose 5TMS BP | 1805.14 | 277 277 0 277 277 | 0 | 0.972 | 0.997 | *NA* | 0.946 | 0.949 |
| | | | 1 | -0.006 | -0.009 | *NA* | 0.028 | 0.000 |
| | | | 2 | -0.034 | -0.018 | *NA* | -0.034 | 0.001 |
| | | | 3 | 0.025 | 0.013 | *NA* | 0.019 | 0.017 |
| | | | 4 | 0.041 | 0.029 | *NA* | 0.042 | 0.016 |
| | | | 5 | *NA* | *NA* | *NA* | 0.011 | 0.024 |
| Citric acid 4TMS | 1812.45 | 375 375 375 375 0 | 0 | 0.748 | 0.765 | 0.804 | 0.912 | *NA* |
| | | | 1 | 0.039 | 0.040 | 0.022 | 0.010 | *NA* |
| | | | 2 | 0.192 | 0.174 | 0.161 | 0.082 | *NA* |
| | | | 3 | 0.010 | 0.011 | 0.005 | *NA* | *NA* |
| | | | 4 | 0.014 | 0.013 | 0.012 | *NA* | *NA* |
| RI 1823 | 1823.07 | 329 329 0 0 329 | 0 | 0.453 | 0.485 | *NA* | *NA* | 0.546 |
| | | | 1 | 0.051 | 0.029 | *NA* | *NA* | -0.002 |
| | | | 2 | 0.393 | 0.400 | *NA* | *NA* | 0.409 |
| | | | 3 | 0.031 | 0.014 | *NA* | *NA* | 0.029 |
| | | | 4 | 0.070 | 0.068 | *NA* | *NA* | 0.030 |
| | | | 5 | 0.013 | *NA* | *NA* | *NA* | *NA* |
| RI 1834 | 1833.51 | 217 217 0 0 0 | 0 | 0.921 | 0.930 | *NA* | *NA* | *NA* |
| | | | 1 | 0.038 | 0.017 | *NA* | *NA* | *NA* |
| | | | 2 | 0.036 | 0.050 | *NA* | *NA* | *NA* |
| | | | 3 | -0.001 | *NA* | *NA* | *NA* | *NA* |
| | | | 4 | 0.011 | *NA* | *NA* | *NA* | *NA* |
| Mannose [-H2O] 4TMS MP | 1838.31 | 217 217 0 0 0 | 0 | 0.559 | 0.564 | *NA* | *NA* | *NA* |
| | | | 1 | 0.418 | 0.421 | *NA* | *NA* | *NA* |
| | | | 2 | 0.023 | 0.022 | *NA* | *NA* | *NA* |
| RI 1883 | 1882.7 | 103 0 0 0 0 | 0 | 0.934 | *NA* | *NA* | *NA* | *NA* |
| | | | 1 | 0.064 | *NA* | *NA* | *NA* | *NA* |
| Glucose 5TMS BP | 1887.02 | 435 435 435 435 435 | 0 | 0.553 | 0.552 | 0.560 | 0.558 | 0.558 |

Continued on next page

Table S1 – *continued from previous page*

| Metabolite | RI | Ions (M0) | M | 21% $O_2$ | 15% $O_2$ | 10% $O_2$ | 5% $O_2$ | 1% $O_2$ |
|---|---|---|---|---|---|---|---|---|
| | | | 1 | 0.002 | 0.003 | -0.005 | 0.002 | 0.003 |
| | | | 2 | 0.538 | 0.541 | 0.533 | 0.530 | 0.525 |
| RI 1897 | 1896.72 | 0 0 0 0 204 | 0 | *NA* | *NA* | *NA* | *NA* | 0.662 |
| | | | 1 | *NA* | *NA* | *NA* | *NA* | 0.374 |
| RI 1897 | 1896.9 | 204 0 0 204 0 | 0 | 0.629 | *NA* | *NA* | 0.647 | *NA* |
| | | | 1 | 0.351 | *NA* | *NA* | 0.334 | *NA* |
| | | | 2 | -0.009 | *NA* | *NA* | -0.003 | *NA* |
| | | | 3 | 0.018 | *NA* | *NA* | 0.012 | *NA* |
| | | | 4 | 0.000 | *NA* | *NA* | -0.007 | *NA* |
| | | | 5 | 0.013 | *NA* | *NA* | 0.019 | *NA* |
| Glucose 1MEOX 5TMS | 1908.55 | 217 217 160 0 0 | 0 | 0.865 | 0.955 | 0.550 | *NA* | *NA* |
| | | | 1 | 0.130 | 0.078 | 0.045 | *NA* | *NA* |
| | | | 2 | -0.004 | *NA* | 0.453 | *NA* | *NA* |
| | | | 3 | 0.024 | *NA* | *NA* | *NA* | *NA* |
| Lysine 4TMS | 1916.85 | 0 0 0 0 186 | 0 | *NA* | *NA* | *NA* | *NA* | 0.949 |
| | | | 1 | *NA* | *NA* | *NA* | *NA* | -0.005 |
| | | | 2 | *NA* | *NA* | *NA* | *NA* | 0.013 |
| | | | 3 | *NA* | *NA* | *NA* | *NA* | 0.015 |
| | | | 4 | *NA* | *NA* | *NA* | *NA* | 0.030 |
| | | | 5 | *NA* | *NA* | *NA* | *NA* | 0.030 |
| RI 1922 | 1922.17 | 319 319 0 319 0 | 0 | 0.912 | 0.936 | *NA* | 0.927 | *NA* |
| | | | 1 | 0.027 | 0.014 | *NA* | 0.014 | *NA* |
| | | | 2 | 0.058 | 0.048 | *NA* | 0.034 | *NA* |
| | | | 3 | *NA* | -0.010 | *NA* | 0.022 | *NA* |
| | | | 4 | *NA* | 0.017 | *NA* | *NA* | *NA* |
| RI 1929 | 1929.02 | 345 345 345 345 345 | 0 | 0.581 | 0.614 | 0.597 | 0.596 | 0.616 |
| | | | 1 | 0.012 | 0.010 | 0.009 | 0.014 | 0.005 |
| | | | 2 | 0.470 | 0.434 | 0.452 | 0.451 | 0.447 |

Continued on next page

**Table S1 – *continued from previous page***

| Metabolite | RI | Ions (M0) | M | 21% $O_2$ | 15% $O_2$ | 10% $O_2$ | 5% $O_2$ | 1% $O_2$ |
|---|---|---|---|---|---|---|---|---|
| RI 1955 | 1954.81 | 333 333 0 0 0 | 0 | 0.926 | 0.969 | *NA* | *NA* | *NA* |
| | | | 1 | 0.014 | 0.004 | *NA* | *NA* | *NA* |
| | | | 2 | 0.030 | 0.085 | *NA* | *NA* | *NA* |
| | | | 3 | 0.037 | -0.069 | *NA* | *NA* | *NA* |
| | | | 4 | *NA* | 0.011 | *NA* | *NA* | *NA* |
| Glucose 5TMS MP | 1974.11 | 435 435 435 435 435 | 0 | 0.565 | 0.567 | 0.554 | 0.563 | 0.574 |
| | | | 1 | -0.001 | 0.000 | -0.001 | -0.001 | -0.009 |
| | | | 2 | 0.525 | 0.516 | 0.533 | 0.523 | 0.530 |
| RI 1986 | 1986.25 | 217 217 217 217 217 | 0 | 0.857 | 0.872 | 0.863 | 0.877 | 0.825 |
| | | | 1 | 0.136 | 0.126 | 0.123 | 0.111 | 0.148 |
| | | | 2 | *NA* | *NA* | *NA* | *NA* | 0.010 |
| | | | 3 | *NA* | *NA* | *NA* | *NA* | 0.021 |
| RI 1990 | 1989.96 | 333 0 0 0 292 | 0 | 0.550 | *NA* | *NA* | *NA* | 0.589 |
| | | | 1 | 0.013 | *NA* | *NA* | *NA* | 0.003 |
| | | | 2 | 0.437 | *NA* | *NA* | *NA* | 0.460 |
| | | | 3 | 0.003 | *NA* | *NA* | *NA* | *NA* |
| | | | 4 | 0.012 | *NA* | *NA* | *NA* | *NA* |
| RI 2012 | 2012.02 | 243 243 243 243 0 | 0 | 0.537 | 0.531 | 0.541 | 0.537 | *NA* |
| | | | 1 | 0.282 | 0.293 | 0.287 | 0.302 | *NA* |
| | | | 2 | 0.112 | 0.106 | 0.111 | 0.112 | *NA* |
| | | | 3 | 0.056 | 0.046 | 0.055 | 0.061 | *NA* |
| | | | 4 | 0.024 | 0.021 | 0.017 | *NA* | *NA* |
| RI 2023 | 2023.22 | 0 346 0 0 0 | 0 | *NA* | 0.895 | *NA* | *NA* | *NA* |
| | | | 1 | *NA* | 0.021 | *NA* | *NA* | *NA* |
| | | | 2 | *NA* | 0.073 | *NA* | *NA* | *NA* |
| | | | 3 | *NA* | 0.022 | *NA* | *NA* | *NA* |
| Hexadecanoic acid 1TMS | 2042.73 | 313 313 0 0 0 | 0 | 0.762 | 0.779 | *NA* | *NA* | *NA* |
| | | | 1 | 0.021 | 0.008 | *NA* | *NA* | *NA* |

Continued on next page

Table S1 – ***continued from previous page***

| Metabolite | RI | Ions (M0) | M | 21% $O_2$ | 15% $O_2$ | 10% $O_2$ | 5% $O_2$ | 1% $O_2$ |
|---|---|---|---|---|---|---|---|---|
| | | | 2 | 0.094 | 0.102 | *NA* | *NA* | *NA* |
| | | | 3 | 0.031 | 0.020 | *NA* | *NA* | *NA* |
| | | | 4 | 0.083 | 0.070 | *NA* | *NA* | *NA* |
| | | | 5 | -0.010 | 0.003 | *NA* | *NA* | *NA* |
| | | | 6 | 0.024 | 0.024 | *NA* | *NA* | *NA* |
| RI 2070 | 2069.62 | 103 103 0 0 0 | 0 | 0.889 | 0.870 | *NA* | *NA* | *NA* |
| | | | 1 | 0.120 | 0.115 | *NA* | *NA* | *NA* |
| | | | 2 | -0.023 | 0.004 | *NA* | *NA* | *NA* |
| | | | 3 | 0.015 | 0.010 | *NA* | *NA* | *NA* |
| RI 2075 | 2075.49 | 0 103 103 0 0 | 0 | *NA* | 0.889 | 0.870 | *NA* | *NA* |
| | | | 1 | *NA* | 0.124 | 0.101 | *NA* | *NA* |
| | | | 2 | *NA* | -0.024 | 0.008 | *NA* | *NA* |
| | | | 3 | *NA* | 0.011 | 0.022 | *NA* | *NA* |
| RI 2158 | 2157.62 | 282 0 0 0 0 | 0 | 0.920 | *NA* | *NA* | *NA* | *NA* |
| | | | 1 | 0.032 | *NA* | *NA* | *NA* | *NA* |
| | | | 2 | 0.049 | *NA* | *NA* | *NA* | *NA* |
| RI 2163 | 2163.02 | 0 217 0 217 0 | 0 | *NA* | 0.938 | *NA* | 0.891 | *NA* |
| | | | 1 | *NA* | 0.032 | *NA* | 0.039 | *NA* |
| | | | 2 | *NA* | 0.029 | *NA* | 0.062 | *NA* |
| | | | 3 | *NA* | 0.000 | *NA* | 0.028 | *NA* |
| | | | 4 | *NA* | 0.012 | *NA* | *NA* | *NA* |
| RI 2249 | 2248.81 | 0 217 217 0 0 | 0 | *NA* | 0.902 | 0.926 | *NA* | *NA* |
| | | | 1 | *NA* | 0.054 | 0.033 | *NA* | *NA* |
| | | | 2 | *NA* | 0.042 | 0.051 | *NA* | *NA* |
| RI 2268 | 2268.1 | 0 232 232 0 232 | 0 | *NA* | 0.525 | 0.515 | *NA* | 0.528 |
| | | | 1 | *NA* | 0.472 | 0.478 | *NA* | 0.479 |
| | | | 2 | *NA* | *NA* | 0.011 | *NA* | *NA* |
| RI 2473 | 2472.66 | 287 287 287 287 287 | 0 | 0.500 | 0.534 | 0.541 | 0.515 | 0.550 |

Continued on next page

**Table S1 – *continued from previous page***

| Metabolite | RI | Ions (M0) | M | 21% $O_2$ | 15% $O_2$ | 10% $O_2$ | 5% $O_2$ | 1% $O_2$ |
|---|---|---|---|---|---|---|---|---|
| | | | 1 | 0.470 | 0.445 | 0.523 | 0.458 | 0.470 |
| | | | 2 | 0.032 | 0.019 | *NA* | 0.028 | *NA* |
| | | | 3 | *NA* | *NA* | *NA* | 0.017 | *NA* |
| RI 2578 | 2578.32 | 363 363 363 363 0 | 0 | 0.821 | 0.832 | 0.869 | 0.939 | *NA* |
| | | | 1 | 0.052 | 0.033 | 0.003 | 0.006 | *NA* |
| | | | 2 | 0.113 | 0.131 | 0.113 | 0.067 | *NA* |
| | | | 3 | 0.004 | -0.002 | -0.005 | *NA* | *NA* |
| | | | 4 | 0.007 | 0.011 | 0.004 | *NA* | *NA* |
| | | | 5 | 0.016 | *NA* | 0.038 | *NA* | *NA* |
| RI 2865 | 2864.58 | 315 315 315 315 315 | 0 | 0.995 | 0.991 | 1.007 | 1.007 | 0.946 |
| | | | 1 | *NA* | *NA* | *NA* | *NA* | -0.007 |
| | | | 2 | *NA* | *NA* | *NA* | *NA* | 0.057 |
| | | | 3 | *NA* | *NA* | *NA* | *NA* | -0.007 |
| | | | 4 | *NA* | *NA* | *NA* | *NA* | 0.012 |
| RI 3037 | 3037.3 | 258 258 258 258 258 | 0 | 0.529 | 0.548 | 0.534 | 0.571 | 0.599 |
| | | | 1 | 0.299 | 0.274 | 0.270 | 0.251 | 0.185 |
| | | | 2 | 0.113 | 0.123 | 0.122 | 0.125 | 0.114 |
| | | | 3 | 0.044 | 0.046 | 0.057 | 0.054 | 0.046 |
| | | | 4 | 0.019 | 0.020 | 0.018 | 0.007 | 0.026 |
| | | | 5 | *NA* | *NA* | 0.012 | 0.014 | 0.012 |
| | | | 6 | *NA* | *NA* | *NA* | *NA* | 0.035 |
| AMP 5TMS | 3050.94 | 169 169 169 0 169 | 0 | 0.517 | 0.537 | 0.533 | *NA* | 0.633 |
| | | | 1 | 0.306 | 0.278 | 0.279 | *NA* | 0.198 |
| | | | 2 | 0.122 | 0.131 | 0.126 | *NA* | 0.094 |
| | | | 3 | 0.045 | 0.046 | 0.045 | *NA* | 0.060 |
| | | | 4 | *NA* | 0.011 | 0.020 | *NA* | 0.017 |

**Table S2:** All compounds which have been detected as isotopically enriched along with their corrected MIDs of the largest detected fragment after [U-$^{13}$C]glutamine.

| **Metabolite** | **RI** | **Ions (M0)** | **M** | **21% $O_2$** | **15% $O_2$** | **10% $O_2$** | **5% $O_2$** | **1% $O_2$** |
|---|---|---|---|---|---|---|---|---|
| RI 965 | 964.562 | 0 0 131 131 0 | 0 | *NA* | *NA* | 0.950 | 0.940 | *NA* |
| | | | 1 | *NA* | *NA* | 0.007 | -0.001 | *NA* |
| | | | 2 | *NA* | *NA* | 0.004 | 0.003 | *NA* |
| | | | 3 | *NA* | *NA* | 0.003 | 0.008 | *NA* |
| | | | 4 | *NA* | *NA* | 0.021 | 0.040 | *NA* |
| | | | 5 | *NA* | *NA* | -0.001 | -0.005 | *NA* |
| | | | 6 | *NA* | *NA* | 0.021 | -0.001 | *NA* |
| | | | 7 | *NA* | *NA* | *NA* | 0.028 | *NA* |
| Proline 1TMS | 1173.02 | 0 103 103 103 0 | 0 | *NA* | 0.665 | 0.654 | 0.663 | *NA* |
| | | | 1 | *NA* | 0.313 | 0.350 | 0.322 | *NA* |
| | | | 2 | *NA* | 0.013 | *NA* | 0.016 | *NA* |
| | | | 3 | *NA* | 0.010 | *NA* | *NA* | *NA* |
| Proline 2TMS | 1296.65 | 216 216 216 216 216 | 0 | 0.731 | 0.736 | 0.721 | 0.718 | 0.774 |
| | | | 1 | 0.034 | 0.035 | 0.016 | 0.020 | 0.006 |
| | | | 2 | -0.007 | -0.007 | -0.003 | -0.004 | -0.001 |
| | | | 3 | 0.001 | 0.001 | 0.001 | 0.001 | 0.000 |
| | | | 4 | 0.234 | 0.223 | 0.253 | 0.265 | 0.219 |
| | | | 5 | -0.012 | -0.003 | -0.006 | -0.014 | -0.012 |
| | | | 6 | 0.021 | 0.016 | 0.021 | 0.016 | 0.018 |
| Aspartic acid 2TMS | 1419.9 | 160 160 160 0 0 | 0 | 0.629 | 0.644 | 0.621 | *NA* | *NA* |
| | | | 1 | 0.085 | 0.081 | 0.063 | *NA* | *NA* |
| | | | 2 | 0.081 | 0.086 | 0.098 | *NA* | *NA* |
| | | | 3 | 0.210 | 0.189 | 0.221 | *NA* | *NA* |
| Malic acid 3TMS | 1487.87 | 245 245 245 245 245 | 0 | 0.643 | 0.656 | 0.649 | 0.668 | 0.652 |
| | | | 1 | 0.042 | 0.042 | 0.021 | 0.004 | 0.008 |
| | | | 2 | 0.091 | 0.086 | 0.071 | 0.038 | 0.023 |

Continued on next page

**Table S2 – *continued from previous page***

| **Metabolite** | **RI** | **Ions (M0)** | **M** | **21% $O_2$** | **15% $O_2$** | **10% $O_2$** | **5% $O_2$** | **1% $O_2$** |
|---|---|---|---|---|---|---|---|---|
| | | | 3 | 0.059 | 0.064 | 0.103 | 0.186 | 0.290 |
| | | | 4 | 0.191 | 0.175 | 0.179 | 0.120 | 0.030 |
| Pyroglutamic acid 2TMS | 1516.53 | 258 258 258 258 258 | 0 | 0.568 | 0.578 | 0.565 | 0.579 | 0.574 |
| | | | 1 | 0.012 | 0.011 | 0.005 | 0.001 | 0.000 |
| | | | 2 | 0.015 | 0.013 | 0.015 | 0.010 | 0.003 |
| | | | 3 | 0.040 | 0.035 | 0.033 | 0.023 | 0.005 |
| | | | 4 | 0.013 | 0.013 | 0.013 | 0.014 | 0.016 |
| | | | 5 | 0.384 | 0.380 | 0.403 | 0.408 | 0.437 |
| Aspartic acid 3TMS | 1519.22 | 306 306 306 306 306 | 0 | 0.621 | 0.635 | 0.615 | 0.648 | 0.633 |
| | | | 1 | 0.074 | 0.069 | 0.058 | 0.022 | 0.019 |
| | | | 2 | 0.072 | 0.075 | 0.090 | 0.121 | 0.156 |
| | | | 3 | 0.234 | 0.223 | 0.235 | 0.209 | 0.178 |
| | | | 4 | *NA* | *NA* | *NA* | *NA* | 0.010 |
| | | | 5 | *NA* | *NA* | *NA* | *NA* | 0.010 |
| Glutamic acid 2TMS | 1531.48 | 276 276 276 276 276 | 0 | 0.595 | 0.594 | 0.574 | 0.583 | 0.584 |
| | | | 1 | 0.028 | 0.041 | 0.030 | 0.009 | 0.007 |
| | | | 2 | 0.019 | 0.041 | 0.030 | 0.031 | 0.022 |
| | | | 3 | 0.097 | 0.084 | 0.077 | 0.048 | 0.015 |
| | | | 4 | 0.018 | 0.013 | 0.016 | 0.019 | 0.022 |
| | | | 5 | 0.264 | 0.251 | 0.296 | 0.337 | 0.374 |
| | | | 6 | *NA* | *NA* | *NA* | *NA* | 0.010 |
| 2-Hydroxypent-2-endioic acid 3TMS | 1604.4 | 0 347 0 0 0 | 0 | *NA* | 0.592 | *NA* | *NA* | *NA* |
| | | | 1 | *NA* | 0.033 | *NA* | *NA* | *NA* |
| | | | 2 | *NA* | 0.035 | *NA* | *NA* | *NA* |
| | | | 3 | *NA* | 0.087 | *NA* | *NA* | *NA* |
| | | | 4 | *NA* | 0.029 | *NA* | *NA* | *NA* |
| | | | 5 | *NA* | 0.261 | *NA* | *NA* | *NA* |
| Glutamic acid 3TMS | 1616.94 | 348 348 348 348 348 | 0 | 0.582 | 0.599 | 0.580 | 0.596 | 0.596 |

Continued on next page

**Table S2 – *continued from previous page***

| Metabolite | RI | Ions (M0) | M | 21% $O_2$ | 15% $O_2$ | 10% $O_2$ | 5% $O_2$ | 1% $O_2$ |
|---|---|---|---|---|---|---|---|---|
| | | | 1 | 0.032 | 0.030 | 0.018 | 0.001 | 0.005 |
| | | | 2 | 0.035 | 0.037 | 0.032 | 0.022 | 0.006 |
| | | | 3 | 0.093 | 0.092 | 0.078 | 0.044 | 0.008 |
| | | | 4 | 0.011 | 0.009 | 0.011 | 0.013 | 0.016 |
| | | | 5 | 0.283 | 0.270 | 0.324 | 0.373 | 0.425 |
| RI 1627 | 1627.07 | 172 172 172 0 0 | 0 | 0.619 | 0.597 | 0.599 | *NA* | *NA* |
| | | | 1 | 0.059 | 0.053 | 0.048 | *NA* | *NA* |
| | | | 2 | 0.350 | 0.347 | 0.355 | *NA* | *NA* |
| | | | 3 | *NA* | -0.017 | *NA* | *NA* | *NA* |
| | | | 4 | *NA* | 0.020 | *NA* | *NA* | *NA* |
| N-acetylaspartic acid 2TMS | 1651.78 | 245 245 245 245 245 | 0 | 0.635 | 0.667 | 0.638 | 0.700 | 0.666 |
| | | | 1 | 0.038 | 0.019 | 0.010 | 0.002 | 0.009 |
| | | | 2 | 0.066 | 0.073 | 0.060 | 0.030 | 0.023 |
| | | | 3 | 0.083 | 0.079 | 0.107 | 0.154 | 0.266 |
| | | | 4 | 0.183 | 0.170 | 0.183 | 0.122 | 0.041 |
| | | | 5 | *NA* | *NA* | *NA* | 0.014 | *NA* |
| Xylitol 5TMS | 1718.27 | 319 0 0 0 319 | 0 | 0.949 | *NA* | *NA* | *NA* | 1.010 |
| | | | 1 | 0.052 | *NA* | *NA* | *NA* | *NA* |
| Glutamine 3TMS | 1768.5 | 347 347 347 347 347 | 0 | 0.519 | 0.530 | 0.514 | 0.499 | 0.513 |
| | | | 1 | 0.012 | 0.006 | 0.008 | 0.023 | 0.013 |
| | | | 2 | 0.044 | 0.052 | 0.052 | 0.067 | 0.056 |
| | | | 3 | 0.004 | -0.011 | 0.004 | 0.011 | 0.014 |
| | | | 4 | 0.019 | 0.010 | 0.021 | 0.013 | 0.023 |
| | | | 5 | 0.453 | 0.460 | 0.457 | 0.438 | 0.438 |
| | | | 6 | *NA* | 0.018 | *NA* | *NA* | *NA* |
| Citric acid 4TMS | 1812.45 | 375 375 375 375 0 | 0 | 0.616 | 0.645 | 0.623 | 0.665 | *NA* |
| | | | 1 | 0.048 | 0.043 | 0.022 | 0.024 | *NA* |
| | | | 2 | 0.091 | 0.081 | 0.068 | 0.028 | *NA* |

Continued on next page

**Table S2 – *continued from previous page***

| Metabolite | RI | Ions (M0) | M | 21% $O_2$ | 15% $O_2$ | 10% $O_2$ | 5% $O_2$ | 1% $O_2$ |
|---|---|---|---|---|---|---|---|---|
| | | | 3 | 0.061 | 0.060 | 0.076 | 0.072 | *NA* |
| | | | 4 | 0.150 | 0.133 | 0.134 | 0.077 | *NA* |
| | | | 5 | 0.042 | 0.048 | 0.092 | 0.163 | *NA* |
| RI 1823 | 1823.07 | 0 404 0 0 0 | 0 | *NA* | 0.928 | *NA* | *NA* | *NA* |
| | | | 1 | *NA* | 0.035 | *NA* | *NA* | *NA* |
| | | | 2 | *NA* | 0.035 | *NA* | *NA* | *NA* |
| RI 1920 | 1920.26 | 0 0 362 0 0 | 0 | *NA* | *NA* | 0.593 | *NA* | *NA* |
| | | | 1 | *NA* | *NA* | 0.022 | *NA* | *NA* |
| | | | 2 | *NA* | *NA* | 0.094 | *NA* | *NA* |
| | | | 3 | *NA* | *NA* | 0.017 | *NA* | *NA* |
| | | | 4 | *NA* | *NA* | 0.313 | *NA* | *NA* |
| Pantothenic acid 3TMS | 1983.3 | 0 0 0 291 0 | 0 | *NA* | *NA* | *NA* | 0.941 | *NA* |
| | | | 1 | *NA* | *NA* | *NA* | 0.000 | *NA* |
| | | | 2 | *NA* | *NA* | *NA* | 0.078 | *NA* |
| RI 2024 | 2023.57 | 346 0 0 0 0 | 0 | 0.535 | *NA* | *NA* | *NA* | *NA* |
| | | | 1 | 0.032 | *NA* | *NA* | *NA* | *NA* |
| | | | 2 | 0.105 | *NA* | *NA* | *NA* | *NA* |
| | | | 3 | 0.032 | *NA* | *NA* | *NA* | *NA* |
| | | | 4 | 0.264 | *NA* | *NA* | *NA* | *NA* |
| | | | 5 | -0.002 | *NA* | *NA* | *NA* | *NA* |
| | | | 6 | 0.031 | *NA* | *NA* | *NA* | *NA* |
| RI 2075 | 2075.48 | 0 132 0 132 0 | 0 | *NA* | 0.916 | *NA* | 0.878 | *NA* |
| | | | 1 | *NA* | 0.047 | *NA* | 0.059 | *NA* |
| | | | 2 | *NA* | 0.012 | *NA* | 0.071 | *NA* |
| | | | 3 | *NA* | 0.036 | *NA* | *NA* | *NA* |
| RI 2158 | 2157.57 | 282 282 282 0 0 | 0 | 0.618 | 0.625 | 0.609 | *NA* | *NA* |
| | | | 1 | 0.025 | 0.022 | 0.013 | *NA* | *NA* |
| | | | 2 | 0.088 | 0.081 | 0.071 | *NA* | *NA* |

Continued on next page

**Table S2 – *continued from previous page***

| Metabolite | RI | Ions (M0) | M | 21% $O_2$ | 15% $O_2$ | 10% $O_2$ | 5% $O_2$ | 1% $O_2$ |
|---|---|---|---|---|---|---|---|---|
| | | | 3 | 0.011 | 0.010 | 0.011 | *NA* | *NA* |
| | | | 4 | 0.273 | 0.264 | 0.291 | *NA* | *NA* |
| Octadecanoic acid 1TMS | 2239.24 | 0 341 0 0 0 | 0 | *NA* | 0.883 | *NA* | *NA* | *NA* |
| | | | 1 | *NA* | -0.006 | *NA* | *NA* | *NA* |
| | | | 2 | *NA* | 0.048 | *NA* | *NA* | *NA* |
| | | | 3 | *NA* | 0.031 | *NA* | *NA* | *NA* |
| | | | 4 | *NA* | 0.040 | *NA* | *NA* | *NA* |
| | | | 5 | *NA* | 0.011 | *NA* | *NA* | *NA* |
| RI 2473 | 2472.66 | 0 0 0 287 0 | 0 | *NA* | *NA* | *NA* | 0.517 | *NA* |
| | | | 1 | *NA* | *NA* | *NA* | 0.020 | *NA* |
| | | | 2 | *NA* | *NA* | *NA* | 0.022 | *NA* |
| | | | 3 | *NA* | *NA* | *NA* | 0.012 | *NA* |
| | | | 4 | *NA* | *NA* | *NA* | 0.020 | *NA* |
| | | | 5 | *NA* | *NA* | *NA* | 0.395 | *NA* |
| | | | 6 | *NA* | *NA* | *NA* | 0.007 | *NA* |
| | | | 7 | *NA* | *NA* | *NA* | 0.000 | *NA* |
| | | | 8 | *NA* | *NA* | *NA* | 0.028 | *NA* |
| RI 2578 | 2578.32 | 363 363 363 363 0 | 0 | 0.681 | 0.705 | 0.686 | 0.710 | *NA* |
| | | | 1 | 0.028 | 0.013 | -0.001 | 0.028 | *NA* |
| | | | 2 | 0.070 | 0.079 | 0.067 | 0.049 | *NA* |
| | | | 3 | 0.157 | 0.152 | 0.148 | 0.093 | *NA* |
| | | | 4 | 0.015 | 0.007 | 0.012 | 0.021 | *NA* |
| | | | 5 | 0.061 | 0.062 | 0.116 | 0.124 | *NA* |

**Table S3:** Metabolites, derivatives and ions in Figure 3 (MID variation).

| **[1,2-$^{13}C_2$]glucose** | | |
|---|---|---|
| **Metabolite** | **Derivative** | **$M_0$ $m/z$** |
| Malate | Malic acid 3TMS | 335 |
| *N*-acetylaspartic acid | *N*-acetylaspartic acid 2TMS | 245 |
| Citrate | Citric acid 4TMS | 375 |
| *Unidentified* | RI 2578 | 363 |
| AMP | 5TMS | 169 |

| **[U-$^{13}$C]glutamine** | | |
|---|---|---|
| **Metabolite** | **Derivative** | **$M_0$ $m/z$** |
| Citrate | Citric acid 4TMS | 375 |
| Malate | Malic acid 3TMS | 245 |
| Glutamate | Glutamic acid 2TMS | 276 |
| Glutamate (not shown) | Glutamic acid 3TMS | 348 |
| *N*-acetylaspartic acid | *N*-acetylaspartic acid 2TMS | 245 |

**Table S4:** Metabolites, derivatives and ions in Figure 4 (MID similarity).

| ***N*-acetylaspartic acid ([U-$^{13}$C]glutamine)** | | |
|---|---|---|
| **Metabolite** | **Derivative** | **$M_0$ $m/z$** |
| Malate | Malic acid 3TMS | 335 |
| Aspartate | Aspartic acid 3TMS | 334 |
| *N*-acetylaspartic acid | *N*-acetylaspartic acid 2TMS | 304 |
| Proline | Proline 1TMS | 172 |

| ***N*-acetylaspartic acid ([1,2-$^{13}C_2$]glucose)** | | |
|---|---|---|
| **Metabolite** | **Derivative** | **$M_0$ $m/z$** |
| *N*-acetylaspartic acid | *N*-acetylaspartic acid 2TMS | 245 |
| Citrate | Citric acid 4TMS | 375 |
| Alanine | Alanine 2TMS | 218 |

| **RI 2578 ([U-$^{13}$C]glutamine)** | | |
|---|---|---|
| **Metabolite** | **Derivative** | **$M_0$ $m/z$** |
| RI 2578 | ? | 363 |
| Citrate | Citric acid 4TMS | 375 |

| **RI 2578 ([1,2-$^{13}C_2$]glucose)** | | |
|---|---|---|
| **Metabolite** | **Derivative** | **$M_0$ $m/z$** |
| RI 2578 | ? | 363 |
| Glycerol-3-phosphate | G3P 4TMS | 357 |
| Pyroglutamate | Pyroglutamic acid 2TMS | 214 |

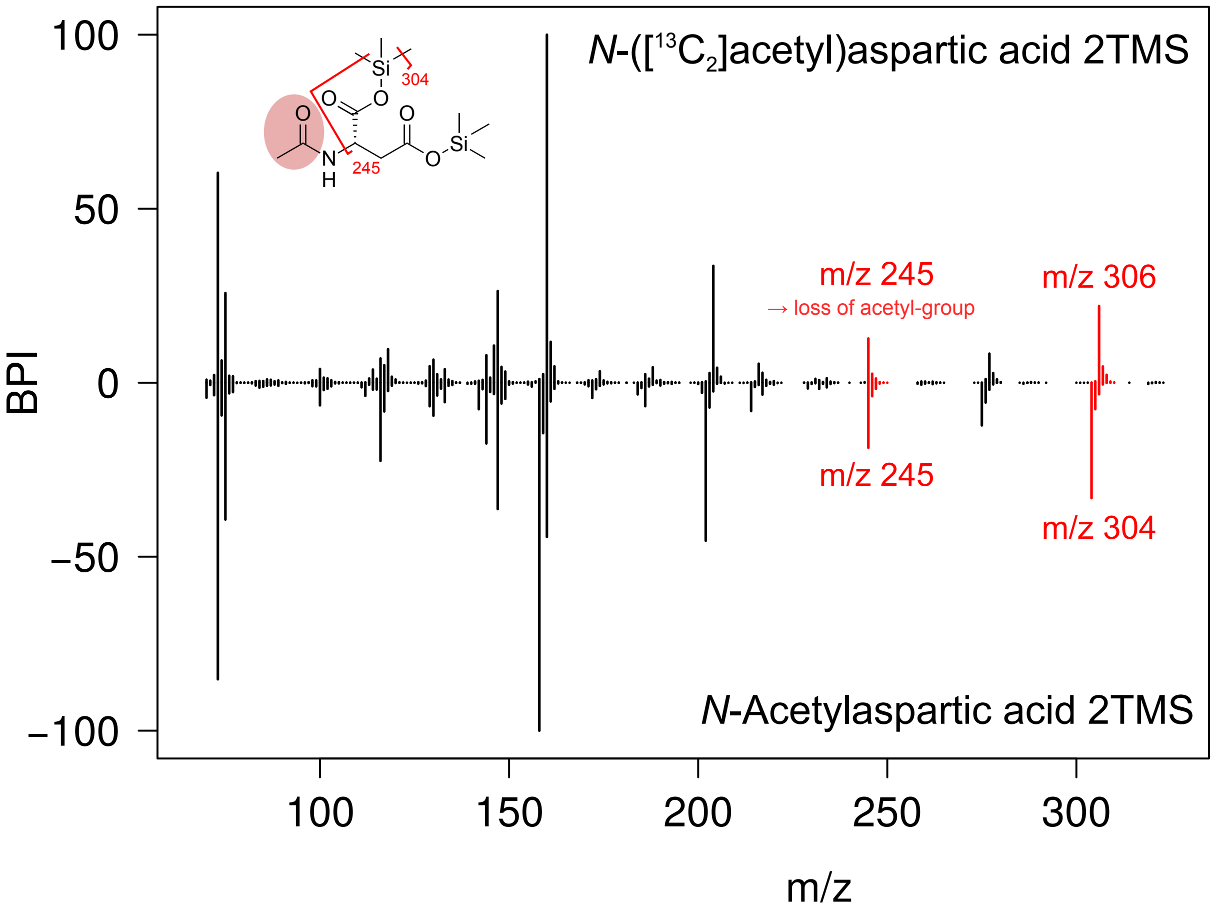


**Figure S5:** EI-MS spectrum of labeled and unlabeled $N$-acetylaspartic acid 2TMS confirming the loss of the acetyl-group in fragment $m/z$ 245.

**Table S6:** siRNA target sequences for gene silencing as part of ON-TARGETplus smart pools (Dharamacon/GEHealthcare).

| Gene symbol | Target sequence |
|---|---|
| *non-targeting* | 5′-UGGUUUACAUGUCGACUAA-3′ |
| | 5′-UGGUUUACAUGUUGUGUGA-3′ |
| | 5′-UGGUUUACAUGUUUUCUGA-3′ |
| | 5′-UGGUUUACAUGUUUUCCUA-3′ |
| NAT8L | 5′-CUUUAAUUCUUGGGACAAA-3′ |
| | 5′-CGGACAUCGAGCAGUACUA-3′ |
| | 5′-GUUUGUACCCUAAGACACA-3′ |
| | 5′-AGUUCGCCGUGGUGCACAA-3′ |

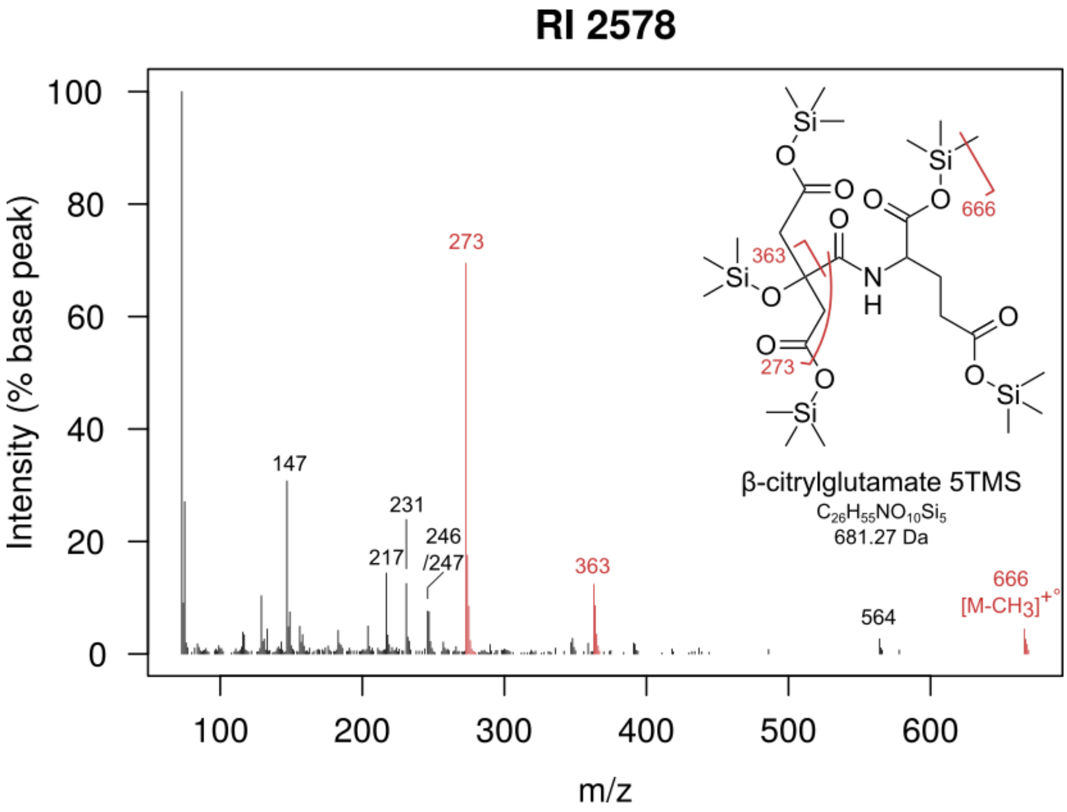


**Figure S7:** EI-MS spectrum with putative structure of $\beta$-citrylglutamate 5TMS and fragmentation of RI 2578.
